# Exploring the diverse binding ability of SARS-CoV-2 variant RBDs to different antibody classes: a computational study

**DOI:** 10.1039/d5ra08084c

**Published:** 2026-03-17

**Authors:** Hoang Linh Nguyen, Nguyen Quoc Thai, Linh Tran, Mai Suan Li

**Affiliations:** a Institute of Fundamental and Applied Sciences, Duy Tan University Ho Chi Minh City 70000 Vietnam nguyenhoanglinh9@duytan.edu.vn; b School of Engineering and Technology, Duy Tan University Da Nang 50000 Vietnam; c Dong Thap University 783 Pham Huu Lau Street, Ward 6 Cao Lanh City Dong Thap 81000 Vietnam nqthai@dthu.edu.vn; d Faculty of Physics, VNU University of Science, Vietnam National University 334 Nguyen Trai Hanoi 10000 Vietnam; e University of Health Sciences, Vietnam National University Ho Chi Minh City Ho Chi Minh City 70000 Vietnam tttlinh@uhsvnu.edu.vn; f Research Center for Discovery and Development of Healthcare Products, Vietnam National University Ho Chi Minh City Ho Chi Minh City 70000 Vietnam; g Institute of Physics, Polish Academy of Sciences al. Lotnikow 32/46 02-668 Warsaw Poland masli@ifpan.edu.pl

## Abstract

The receptor-binding domain (RBD) of the SARS-CoV-2 spike protein is highly immunogenic and structurally dynamic, resulting from continuous evolution and mutations that reshape its antigenic landscape. This study aims to investigate the binding profile between the RBD and four neutralizing antibodies, including class 1 (VIR-7229, S2E12, and OMI-42), class 2 (ZCB11), class 3 (S309), and class 4 (SA55), using computational approaches. These six RBD complexes were subjected to molecular dynamics simulation, and the binding free energy was estimated using the MM-PBSA method. Our results revealed that ZCB11 and S2E12 greatly weakened the binding to Omicron subvariants XBB.1.5, BA.2.86, KP.3, and MV.1, while OMI-42 was evaded by BA.2.86, KP.3, and MV.1. In addition, S309 exhibited a reduced binding affinity to the RBDs of XBB.1.5, BA.2.86, KP.3, and MV.1. Interestingly, glycans contribute approximately 33% to the interface interactions in variants with a single glycan at N343, increasing to 50% with a second glycan at N354. This finding indicates that dual glycans play a crucial role in determining the stability of the antibody–RBD complex. In contrast, SA55 and VIR-7229 demonstrated robust binding across all the studied variants. The viral evolution exhibits a two-tiered strategy to evade antibodies while enhancing ACE2 binding: first, charge-increasing mutations such as Q498R improve ACE2 affinity and repel antibodies like S2E12 and ZCB11; second, neutral mutations, such as F456L/V in KP.3 and MV.1, further weaken antibody binding, as observed in OMI-42. Our results support the charge-centric hypothesis that, although van der Waals interactions are favorable and nearly constant for all variants of a given class of antibodies, electrical interactions determine binding affinity as they vary from variant to variant.

## Abstract 2

A charge-centric thermodynamic framework reveals that SARS-CoV-2 immune evasion is primarily driven by electrostatic repulsion, evolving *via* a two-tiered strategy of charge remodeling and hydrophobic fine-tuning.

## Graphical abstract

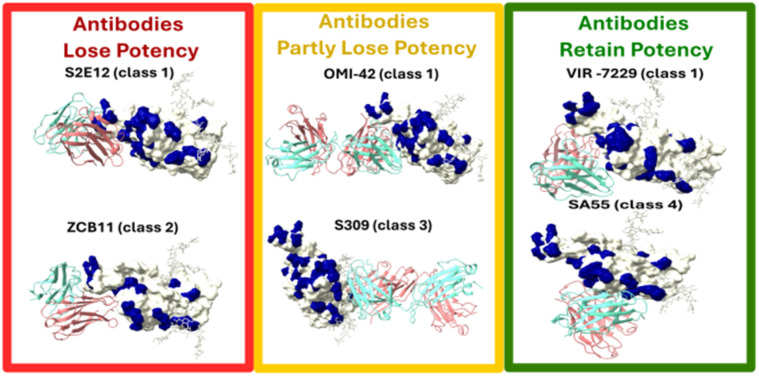

## 1. Introduction

The outbreak of the new coronavirus SARS-CoV-2 ^1^ led to an ongoing pandemic that is called COVID-19.^2^ This pandemic has significantly disrupted global health systems and economic stability worldwide.^3,4^ As of June 2025, more than 777 million individuals have been infected and approximately 7 million deaths have been reported.^5^ Despite the urgency, effective antiviral treatments for COVID-19 remain limited, and novel virus variants exhibit immune evasion.^6,7^ Furthermore, the evolution of SARS-CoV-2 is driven by population immunity,^8^ which poses significant challenges for reliable prediction. Such findings underscore the necessity for intensive research efforts toward the development and optimization of new therapeutic interventions.

The SARS-CoV-2 virion consists of an RNA genome complexed with the nucleocapsid (N) protein, which is enclosed by an outer shell comprising the membrane (M), envelope (E), and spike (S) proteins.^9^ The M and E proteins are embedded within the spherical viral envelope, while the S proteins protrude from the surface into the exterior. The entry of the virus is initiated by anchoring of the S protein to a receptor protein present in the host cell surface.^10–12^ The human protein angiotensin-converting enzyme 2 (ACE2) acts as a receptor for viral entry.^10–12^ In SARS-CoV-2, the S protein is composed of two subunits, S1 and S2. Subunit S1 is responsible for receptor binding, while S2 mediates the fusion process of the viral and host cell membranes.^9^ The receptor binding domain (RBD), which mediates the interactions of the viral spike protein with ACE2, is located in the S1 subunit. The RBD exhibits high immunogenicity and structural dynamics, with ongoing evolution *via* mutations continually reshaping its antigenic landscape. Therefore, RBD is an intriguing target for both natural and therapeutic antibodies.

Antibodies targeting the SARS-CoV-2 spike (S) protein present diverse binding sites, as demonstrated by experimental studies.^13,14^ These antibodies bind to the N-terminal domain (NTD) and RBD at sites distinct from the ACE2 binding region,^13^ or the RBD at sites overlapping with the ACE2 binding site.^15^ Rogers *et al.* reported that the most potent neutralizing antibodies target RBD regions overlapping with the ACE2 binding site, effectively blocking the spike protein's interaction with human ACE2 and neutralizing the virus.^16^ Consequently, the RBD is a key target for antibody development. Neutralizing antibodies targeting the RBD are mainly classified into four major groups, based on their epitopes.^17^ While class 1 and 2 epitopes directly block the ACE2-binding site, class 3 antibodies bind to the outside of the ACE2-binding region, and also bind to RBDs regardless of their ‘up’ and ‘down’ conformations, and class 4 antibodies target a cryptic epitope outside the receptor-binding motif.^17,18^ This diversity of antibody classes drives viral mutations to evade binding, emphasizing the necessity of understanding viral escape pathways at the molecular level to facilitate the development of more effective antibody-based therapeutics.

Recently emerging Omicron sub-variants have demonstrated enhanced immune evasion capabilities^19^ alongside increased ACE2 binding affinity.^20^ While both factors are critical drivers of SARS-CoV-2 evolution,^21^ evidence suggests that immune evasion pressure exerts a more dominant influence on the evolutionary trajectory.^21^ Notably, the BA.2.86 lineage and its derivatives exhibit robust immune escape phenotypes,^22–25^ indicating that the virus retains significant evolutionary potential to generate highly contagious variants. To elucidate the molecular determinants of this resistance, we conducted molecular dynamics (MD) simulations and MM-PBSA analyses to calculate the binding free energies of RBDs from diverse SARS-CoV-2 variants against representative antibodies from four major classes: class 1 (VIR-7229, S2E12, OMI-42), class 2 (ZCB11), class 3 (S309), and class 4 (SA55). Significantly, this work advances the field by establishing a “charge-centric hypothesis” that explicitly separates electrostatic repulsion from van der Waals contact preservation. Through this framework, we identify a “two-tiered evolutionary strategy”: a primary mechanism driven by electrostatic remodeling (tier 1) to repel antibodies, followed by hydrophobic fine-tuning (tier 2) to destabilize residual binding. In other words, our charge-based hypothesis states that electrostatic forces are more favorable than van der Waals forces for good binders, while they are less favorable than van der Waals forces for poor binders. It is no secret that for antibodies with high binding affinity, electrostatic interactions contribute more negatively to the binding free energy than van der Waals interactions,^26–28^ which are more favorable than van der Waals forces. The novelty of our hypothesis is that, thanks to a larger dataset, we showed that, in cases of weak binding, electrostatic interactions become less favorable than their van der Waals counterparts.

Furthermore, we quantify the enthalpic contribution of individual glycan moieties, revealing their dual role in shielding and stabilizing S309 antibody interfaces. This systematic multiscale approach provides critical insights into how the evolution of SARS-CoV-2 is driven by electrostatic repulsion as a primary mechanism of evasion.

## 2. Materials and methods

### 2.1 Initial preparation of the structures

Initial structures for MD simulations were retrieved from the Protein Data Bank (PDB). The class 3-S309 antibody–RBD complexes include PDB IDs 7R6W^29^ for wild type (WT), 7YAD^30^ for Omicron BA.1 and XBB.1.5, and 8Y6A^31^ for BA.2.86, KP.3, and MV.1 variants. The class 4 SA55 antibody-Omicron BA.1 RBD complex was obtained from PDB ID 7Y0W,^32^ the class 1 OMI-42 antibody-RBD complex was from PDB ID 8CBF,^33^ the class 2 ZCB11 antibody-Omicron BA.1 RBD complex was from PDB ID 7XH8,^34^ and the class 1 S2E12 antibody-RBD complex was from PDB ID 7K45.^35^ The class 1-VIR-7229 antibody–RBD complexes include PDBs ID 9AU1 ^36^ for WT, BA.1, and XBB.1.5, and 9ASD^36^ for BA.2.86, KP.3, and MV.1 variants. For antibody–RBD complexes lacking PDB structures, variant-specific RBDs were generated by introducing mutations using the CHARMM-GUI webserver.^37,38^ Given the varying resolutions of the initial PDB structures, we performed a control simulation to validate our results. Specifically, to investigate the influence of the initial conformation, the BA.1 variant was constructed by introducing point mutations into the high-resolution S309-WT crystal structure (PDB ID: 7R6W, 1.83 Å) using CHARMM-GUI, rather than using the lower-resolution cryo-electron microscopy structure.

### 2.2 Simulation setups

**Fig. 1:**
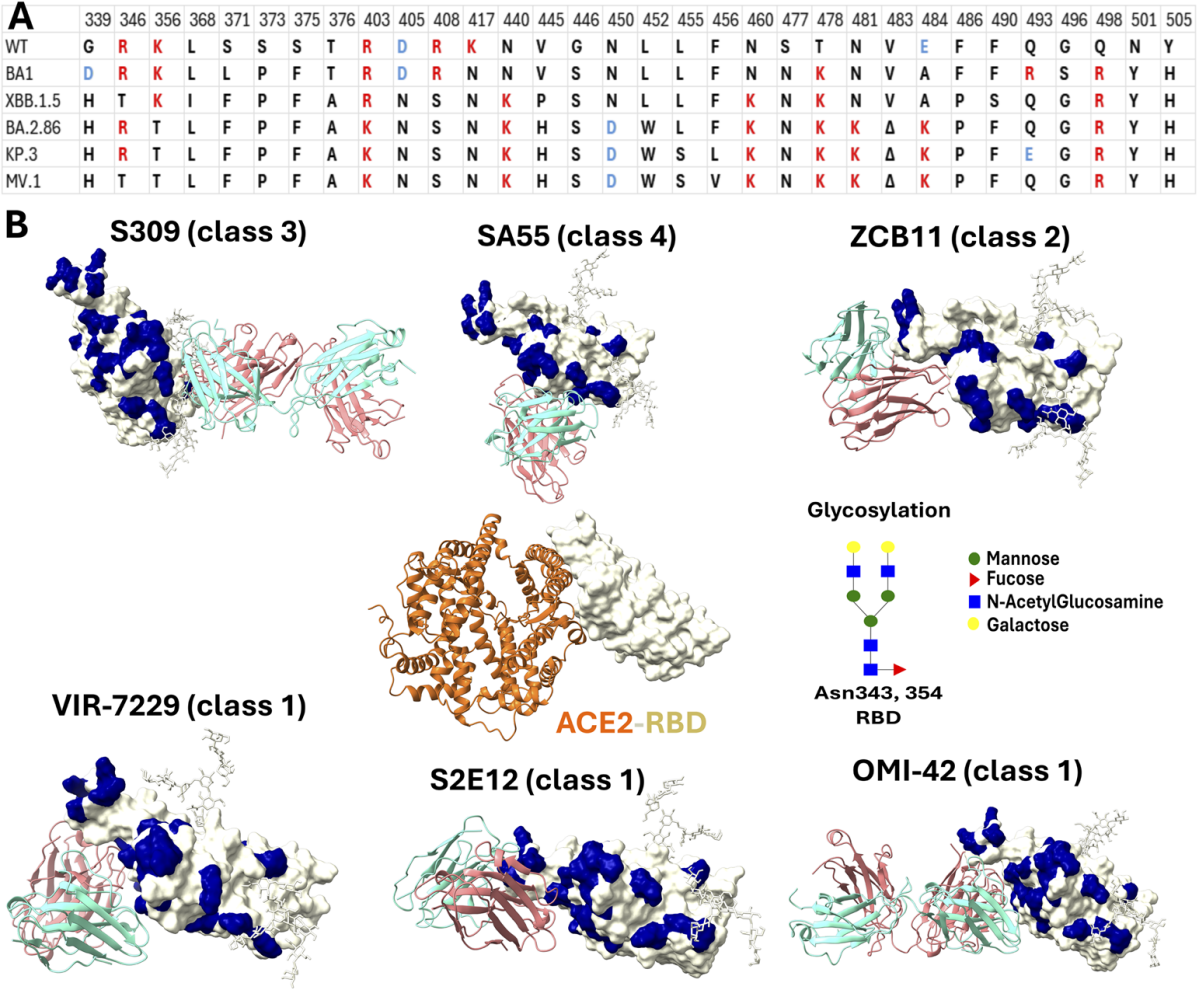
Mutation landscape and structural systems. (A) Amino acid substitutions in the SARS-CoV-2 RBD across the studied variants. Residues are color-coded by net charge: red (positive), blue (negative), and black (neutral). *Δ* Denotes a deletion. (B) Initial structures of the RBD–antibody complexes compared to that of the RBD–ACE2 complex. Mutation positions on the RBD are highlighted in navy. The ACE2-RBD structure (PDB ID 6LZG^44^) is included as a reference for the receptor-binding motif. Representative *N*-glycans at residues N343 and N354 are shown as sticks.

In this study, the parameters for protein and glycan molecules were obtained from the AMBER19SB and GLYCAM 06j forcefields, respectively.^39,40^ The RBD–antibody complexes were simulated within a rectangular box using the OPC water model.^41^ In our study, we utilized the rectangular box option in CHARMM-GUI, maintaining a distance of 10 Å between the RBD protein-antibody complex and the box surface. Consequently, the box dimensions vary depending on the specific RBD–antibody system, ranging from approximately 12.6 nm × 12.6 nm × 12.6 nm to 14.0 nm × 14.0 nm × 14.0 nm. This results in protein concentrations of approximately 0.61 mM to 0.83 mM, calculated at one protein molecule per box. Na^+^ and Cl^−^ ions were added to achieve a physiological concentration of 0.15 M. Protonation states of titratable residues (Asp, Glu, and His) were determined using the CHARMM-GUI webserver to estimate p*K*_a_ at pH 7.0. Standard protonation states were assigned based on these predictions, without custom adjustments required for our systems. In addition, glycan molecules are attached to N343 of the RBD. In the case of BA.2.86, KP.3, and MV.1, the experiment determined that these variants have one more glycan at the N354 residue.^42^ In this work, the *N*-glycan models attached to residues N343 and N354 for specific variants were constructed based on the study by Mehdipour *et al.*^43^ The glycan topologies and parameters were generated using the CHARMM-GUI Glycan Reader and Modeler and described by the GLYCAM06j force field, which is fully compatible with the AMBER19SB protein force field used in our simulations. The schematics for the initial structures are presented in Fig. 1.

### 2.3 Simulation protocol

The solvated systems were minimized by the steepest descent algorithm. Then, the systems were equilibrated in the NVT ensemble at 300 K, followed by the NPT ensemble at 300 K and 1 atm for 0.5 ns and 5 ns, respectively. The temperature of 300 K was chosen because AMBER19SB is parameterized and thoroughly tested at 300 K.^39^ The v-rescale^45^ and c-rescale^46^ algorithms were used to keep constant temperature and pressure, respectively. In the equilibration stages, C-alpha atoms were restrained using the harmonic potential with a spring constant of 1000 kJ mol^−1^ nm^−2^. Finally, for each antibody–RBD variant complex, we performed six independent production MD runs, each of 200 ns duration. To ensure complete independence of trajectories, the simulations were initiated from the same equilibrated structure, but with different random velocities of all atoms. This standard protocol allows us to achieve statistical robustness of independent replicas. The last 100 ns of each trajectory was extracted for data analysis.

### 2.4 Equilibration of RBD–antibody complexes

Fig. S1 shows that the WT RBD–antibody complexes reach equilibrium within 100 ns, as the RMSD obtained from six independent trajectories saturates after this time (the same is true for mutations). To ensure that the 200 ns is sufficient to obtain the binding free energy, for example, we extended the simulations for the S309-WT RBD system to 400 ns. The time dependence of RMSD for this system is shown in Fig. S2, indicating a stable sampling of the bound state ensemble after the first 100 ns. We performed MM-PBSA analysis for the 100–200 ns, 100–400 ns, and 200–400 ns windows of the S309-WT RBD system. Within the error bars, the binding free energy Δ*G* for these windows is the same (Table S1), demonstrating that 200 ns runs are sufficient to reach equilibrium and obtain reliable estimates. Therefore, snapshots obtained in the 100–200 ns range were used for data analysis.

To evaluate the potential impact of different starting resolutions, we calculated the MM-PBSA binding free energy for the S309-BA.1 complex using a model generated from the high-resolution WT crystal structure (PDB ID: 7R6W). The resulting binding free energy for this modeled BA.1 system (−34.33 ± 5.87 kcal mol^−1^) is statistically indistinguishable from that of the Cryo-EM BA.1 structure used in our main study (−34.17 ± 6.41 kcal mol^−1^) (Table S4). We noted differences in individual electrostatic and polar solvation terms between the two systems (Table S4), but their sum remained equivalent. These component-level differences are attributed to the different boundaries of the deposited structures rather than the quality of the resolution: the X-ray structure (7R6W) includes RBD residues 333–527, whereas the Cryo-EM structure (7YAD) covers residues 332–516. The different terminal residues contribute to shifts in the electrostatic and solvation penalties that are compensatory in nature, ultimately yielding a consistent total binding free energy. This confirms that our protocol effectively mitigates the influence of initial structural resolution.

### 2.5 Data analysis

The binding free energy between the RBD and antibody was estimated using the MM-PBSA method.^47^ The binding free energy is decomposed into components as follows: Δ*G* = Δ*E*_MM_ + Δ*E*_Polar_ + Δ*E*_Non-polar_ − *T*Δ*S*. The Δ*E*_MM_ is the non-bonded interaction contribution between two molecules, including electrostatic and van der Waals energies, without a cutoff. The Δ*E*_Polar_ and Δ*E*_Non-polar_ are polar solvation and non-polar solvation energy, respectively. The Delphi package was used to calculate polar solvation.^48^ The Δ*E*_Non-polar_ was obtained from the equation Δ*E*_Non-polar_ = *γ*ΔSASA where *γ* = 0.0052 kcal mol^−1^ Å^−2^ and SASA is the solvent accessible surface area. The entropy Δ*S* was estimated using the interaction entropy method.^49^ The non-bonded interaction energy between the RBD and antibody was calculated without a cutoff. The entropic contribution was calculated from the equation 
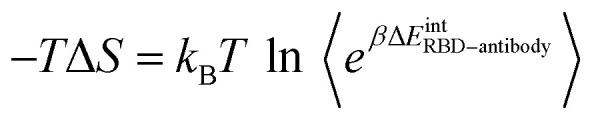
, where *β* = 1/*k*_B_*T*, *k*_B_ is the Boltzmann constant, Δ*E*^int^_RBD–antibody_ = *E*^int^_RBD–antibody_ − 〈*E*^int^_RBD–antibody_〉 is the fluctuation of the RBD–antibody interaction energy around the average energy 〈*E*^int^_RBD–antibody_〉, 〈…〉 represents the average over the collected snapshots. The side-chain contact between two residues is formed when the distance between the centers of mass is smaller than or equal to 6.5 Å.

Per-residue interaction energies were calculated using an in-house program, utilizing force field parameters extracted from the system topologies. Non-bonded energies were computed between each residue in one protein and all residues in the other protein, without applying a cutoff distance. To explicitly evaluate the role of glycans, we partitioned this non-bonded interaction energy into two distinct components: the interaction between the antibody and the RBD protein, and the interaction between the antibody and the *N*-glycan moiety. Both contributions were computed using the same force field parameters and infinite cutoff settings. Calculations were performed on snapshots sampled from the final 100 ns of each trajectory. The buried surface areas (BSA) were calculated as half of the difference between the sum of the SASA of the isolated RBD and antibody and the SASA of the complex: BSA = 0.5 × (SASA_RBD_ + SASA_antibody_ − SASA_complex_).

### 2.5 Statistical analysis

The final binding free energy for each system is reported as the arithmetic mean derived from six independent trajectories. The uncertainties are presented as standard deviations (SD) to reflect the variation of the results across the independent replicas.

## 3. Results and discussion

### 3.1 Binding sites of the SARS-CoV-2 RBD in complex with antibodies

**Fig. 2:**
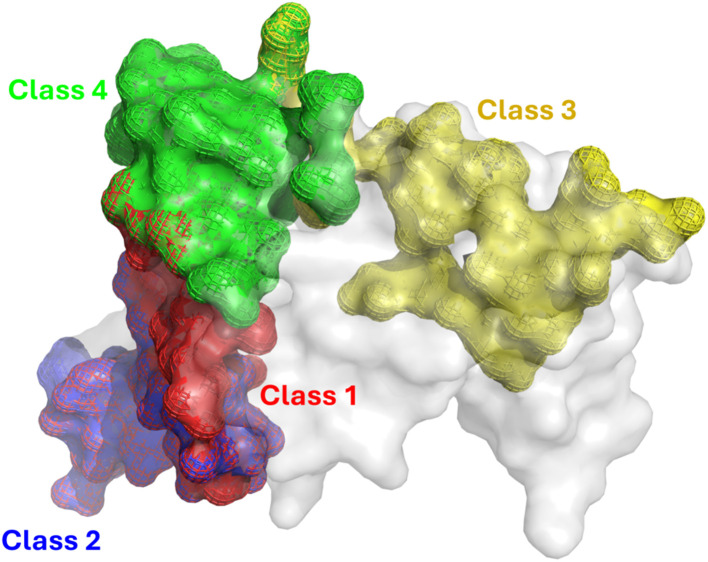
Structural comparison of epitope footprints for different antibody classes on the SARS-CoV-2 RBD. The RBD surface is rendered in grey. Binding interfaces are highlighted with solid colors overlaid by mesh outlines to visualize the spatial distribution of epitopes.

Structural superposition reveals that the epitopes of class 1 and 2 antibodies are largely overlapping and coincide with the receptor binding motif (RBM) (Fig. 2). In contrast, the class 3 epitope is spatially distinct, located on the RBD core distal to the RBM. Class 4 occupies an intermediate region bridging the class 3 site and the RBM. Quantitative analysis of the interface properties (Table S1) indicates that class 1 antibodies engage over the most extensive contact area, exhibiting the highest number of contact residues and the largest buried surface area. Notably, the class 1 epitope is electrostatically distinct, carrying the highest net charge (+3e) compared to classes 2, 3, and 4 (+1e, +2e, and 0, respectively). Furthermore, while the mean hydrophobicity is comparable among classes 2, 3, and 4, it is significantly lower for class 1 (Table S1). These distinct physicochemical profiles highlight the diverse recognition mechanisms employed by different antibody classes, which likely govern their differential susceptibility to viral escape mutations.

Specifically, the binding interfaces of class 1 antibodies VIR-7229, S2E12, and OMI-42 are primarily anchored by two residue clusters: 400–425 and 450–500 (Fig. 2, and S3–S5), consistent with experimental observations.^36,50^ Generally, newer RBD variants establish more extensive and diverse contact networks with VIR-7229 and OMI-42 compared to older lineages (Fig. S3 and S5). In contrast, S2E12 engages strictly with the 450–500 cluster while making minimal contact with the 400–425 region. Since the 400–425 segment exhibits a significantly lower mutation frequency than the hypervariable 450–500 region (Fig. 1A), this specific reliance renders S2E12 highly susceptible to immune escape. Crucially, although newer variants, such as BA.2.86, KP.3, and MV.1, exhibit increased contact density across diverse regions (Fig. S4), this structural expansion does not translate into improved binding affinity (Table 1). This disparity is evident in the divergence of neutralization trends: while VIR-7229 retains potency against novel variants, S2E12 loses efficacy significantly.

**Table 1:** Experimental data for the binding affinities (*K*_D_, nM) of the four antibody classes to the RBD. For studies that report IC50 values, the corresponding *K*_D_ values were calculated from the experimental IC50 values

Antibody	WT	BA.1	XBB.1.5	BA.2.86	KP.3	MV.1
S309	0.81,^51^ 2.21,^52^ 0.2 ± 0.1,^53^ 0.3 ^54^	8.22,^52^ 0.9,^54^ 10.98 ^55^	1.6 ± 0.7 ^53^	>62.84,^56^ >103,^23^ >130 ^57^		
SA55	0.36,^58^ 0.14 ^23^		0.89,^58^ 0.72 ^23^	0.12 ^23^	>240,^59^ 0.04,^60^ 0.22 ^61^	
ZCB11	0.15 ^62^	0.14 ^62^	>41.10 ^62^	>41.10 ^62^		
VIR-7229	0.2 ^36^	0.4 ^36^	0.1 ^36^	0.3 ^36^		
S2E12	2.1 ^54^	44 ^54^	Not determined^54^	>120 ^56^		
	0.02 ^56^	39.3 ^56^	>120 ^56^			
OMI-42	0.25,^58^ 0.678 ^36^	0.24 ^36^	0.45,^36^ 0.21,^58^ 0.29 ^63^	0.44,^36^ 0.36 ^63^		

For class 2 antibody ZCB11, binding occurs at RBD regions 410–425 and 450–505 (Fig. S6), with the 475–500 region being critical, though its 400–425 contacts are less extensive than those of VIR-7229 and OMI-42. Overall, the contact network remains largely conserved across RBD variants; notably, the KP.3 and MV.1 lineages exhibit even stronger contacts in the 493–505 region relative to the WT.

Class 3 antibody S309 relies heavily on glycan interactions, alongside RBD regions 333–350 and 440–450, highlighting the key role of glycans in its binding (Fig. S7). The 440–450 region, like 400–425, has a low mutation frequency, suggesting S309 is less affected by mutations in the 450–500 hotspot. Notably, the MV.1 variant establishes additional contacts in the 319–333 region, supplementing the canonical interactions at residues 333–350 and 440–450.

Finally, the class 4 antibody SA55 targets a diverse region involving glycans and multiple discontinuous segments 373–376, 400–410, and 494–505 of RBD (Fig. S8). As these regions contain highly conserved residues 373–375, 405, 408, 498, 501, and 505 across all studied variants (Fig. 1A), SA55 likely maintains broad neutralization potency across distinct viral lineages. Notably, the XBB.1.5 and BA.2.86 variants establish unique additional contacts in the 479–484 region. However, experimental data indicate that this expanded interaction footprint does not translate into a binding advantage for XBB.1.5 and BA.2.86 compared to other variants (Table 1).

### 3.2 Binding free energy between antibodies and RBD of SARS-CoV-2 variants

Given that changes in contact footprints alone do not fully account for the divergent neutralization potencies against novel SARS-CoV-2 variants, we proceeded to analyze the binding free energy using the MM-PBSA method. The remarkable stability of both the van der Waals interaction and the non-polar solvation energy (Table S3), combined with the residue contact profiles (Fig. S3–S8), confirms that the structural integrity of the antibody–RBD interface remains intact, even in heavily mutated lineages such as KP.3 and MV.1. Consequently, the observed reduction in total binding free energy is driven primarily by unfavorable shifts in the electrostatic component.

The binding sites of antibodies in class 1 and class 2 overlap with ACE2 (Fig. 1). In contrast, the class 3 antibody S309 binds at a distinct site that does not overlap with ACE2 and is uniquely positioned near RBD glycans. The binding site of class 4 antibody SA55 is adjacent to the ACE2 binding site. The mutation positions in variants of the RBD concentrate in the binding regions with classes 1, 2, and 4, but to a lesser extent in class 3. The mutations have a mild impact on the ACE2 binding affinity.^64^ The overlap of the binding site of several antibody classes and ACE2 pressures the virus to not only optimize ACE2 binding but also demolish the antibody binding. Therefore, we investigated the binding free energy of antibodies and variants of viral RBD to unravel the antibody resistance ability of the SARS-CoV-2 virus.

**Fig. 3:**
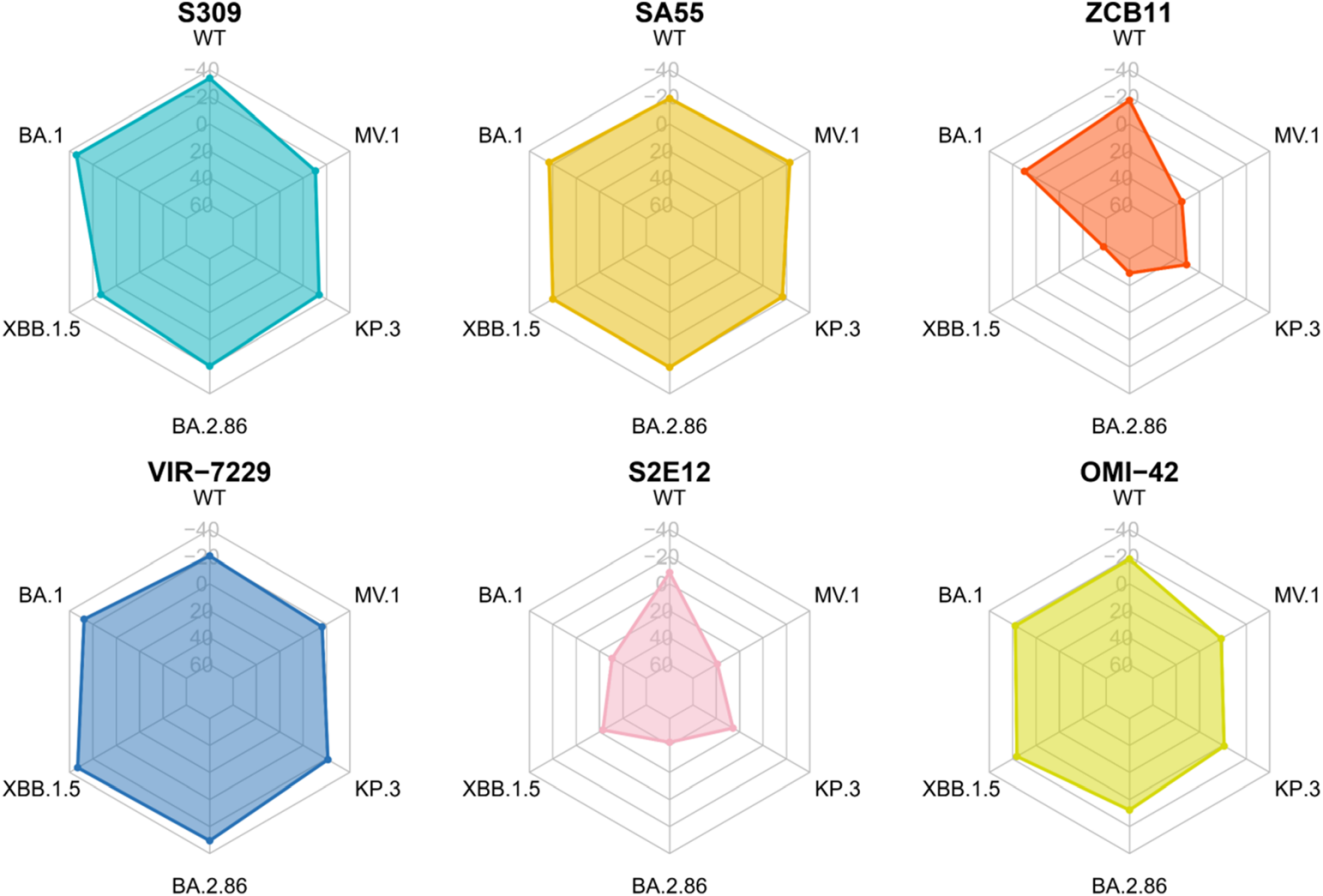
Average binding free energy Δ*G* (kcal mol^−1^) obtained from the MM-PBSA analysis for RBD–antibody complexes.

Although ZCB11 has been shown to remain potent against the wild-type and certain Omicron subvariants,^34^ its binding affinity to Omicron RBDs is notably weakened.^34,65^ Furthermore, another study reported a complete loss of neutralizing ability for this antibody against the Omicron BA.1 variant.^66^ The potency of ZCB11 is drastically decreased with BA.5 and XBB.1.5 variants.^67^ The results of the MM-PBSA calculations in this work show that the binding free energies of ZCB11 with the RBD of the Omicron subvariants are dramatically decreased (Fig. 3; detailed numerical values are provided in Table S3), which is primarily driven by electrostatic interactions. The electrostatic contributions in the Omicron subvariants even elevate to positive values, indicating repulsive forces.

The S2E12 antibody, although exhibiting potency against WT and Omicron BA.1, loses neutralizing activity against Omicron subvariants BA.2, XBB.1.5, and BA.2.86.^56,68^ In this work, the WT RBD has −8.48 kcal mol^−1^ binding free energy to S2E12. However, the calculated binding free energies for S2E12 with Omicron subvariants are markedly weaker than for the WT, with several yielding positive values (Fig. 3), signifying unfavorable interactions and predicted loss of binding. This aligns with experimental evidence of S2E12 evasion by these variants.

The potency of S309 against Omicron subvariants is not decreased from WT except BA.2.86 ^56^ and exhibits further loss of efficacy against JN.1 derived variants.^23,59^ However, the work of Duan *et al.* suggests that S309 also has slightly decreased affinity to BA.1 compared to WT,^69^ which is in line with the results reported by Afzal *et al.*^70^ Our MM-PBSA binding energies (Fig. 3) show a substantial decrease in S309-RBD affinity across Omicron subvariants XBB.1.5, BA.2.86, KP.3, and MV.1. Nevertheless, the energies for recent subvariants such as KP.3 and MV.1 remain negative, implying residual potency without complete evasion, aligning with the experimental data^69,70^ and partly aligning with the results of Li *et al.*^59^ Notably, our MM-PBSA binding energies for S309 align with recent MM-GBSA calculations by Chong *et al.*,^71^ who reported values equivalent to ours. Our MM-PBSA results for S309 are consistent with previous computational studies on its derivative, sotrovimab, which showed robust binding affinity to Omicron variants.^72^ This consistency supports the reliability of our predicted escape trends.

The potency of the SA55 antibody is not weakened against WT and Omicron subvariants, including BA.2.86,^23,32,73^ but is slightly weakened in more novel variants such as LF.1 and LF.7.2.1.^24^ This observation indicates the broad neutralizing activity of this antibody. In this work, the MM-PBSA binding energies of this antibody and RBDs are virtually the same for all variants (Fig. 3). These findings indicate that SA55 effectively counters viral escape across all examined variants, aligning closely with the experimental evidence.

Experiments reveal that VIR-7229 has potent activity against SARS-CoV-2 variants WT, XBB.1.5, BA.2.86, and KP.3.1.1,^36,58^ but the potency diminished slightly in variants such as MC.10.1 and NP.1.^24^ In this study, VIR-7229 maintains binding free energies to the RBD of Omicron subvariants that are equivalent to or more favorable than those to the WT strain (Fig. 3), consistent with the experimental data, demonstrating its robust inhibition of all examined variants.

For OMI-42, experimental evidence indicates effective inhibition of WT through XBB.1.6 and BA.2.86, but evasion by JN.1 and KP.3.^58,74^ Our MM-PBSA results mirror this trend, with binding energies progressively weakening from WT to MV.1, reflecting heightened evasion by newer variants and culminating in loss of affinity against KP.3 and MV.1, in line with the observed experimental escape by JN.1 and KP.3.^74^

In summary, for each antibody, our MM-PBSA results align with the experimental data. Specifically, SA55 and VIR-7229 retain potent binding to all SARS-CoV-2 variants studied. S309 and OMI-42 exhibit reduced binding affinities to BA.2.86, KP.3, and MV.1, while S2E12 and ZCB11 exhibit strongly reduced affinity to Omicron variant RBDs.

**Fig. 4:**
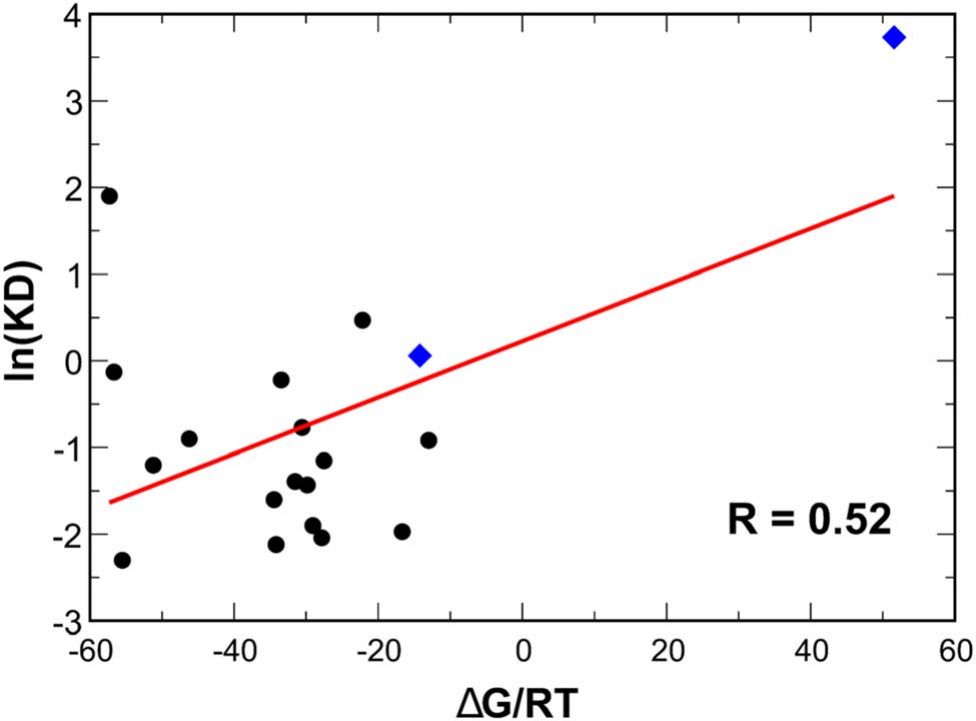
Correlation between Δ*G*/*RT* obtained from the MM-PBSA analysis, where *RT* = 0.5962 kcal mol^−1^ at *T* = 300 K, and the natural logarithm of the experimental *K*_D_. Blue diamonds represent data points of S2E12.

Quantitative correlation analysis between the dimensionless binding free energy Δ*G*/*RT*, where *RT* = 0.5962 kcal mol^−1^ at *T* = 300 K, and the natural logarithm of experimental dissociation constant *K*_D_ (Table S5) yields an overall Pearson coefficient of 0.52 (Fig. 4), indicative of moderate agreement.^75^ Notably, the correlation is strongly driven by the distinct separation between the high-affinity group and the escape variant (S2E12-Omicron). The reduction in correlation upon excluding S2E12 does not reflect a methodological failure but rather highlights the method's primary strength as a binary classifier for viral escape.

Similar to other computational methods, MM/PBSA faces challenges in quantitatively ranking antibodies within the narrow energy window of high-affinity binders due to inherent statistical fluctuations in large protein–protein interfaces; however, it successfully captures the significant shift in the binding free energy associated with resistance. Specifically, the method correctly predicted the transition from binding (negative Δ*G*) to non-binding (positive Δ*G*) for the S2E12 escape mutant.

Furthermore, the correlation is inevitably impacted by experimental heterogeneity. The reference *K*_D_ values were collated from multiple independent studies from different labs utilizing different assays and conditions, introducing experimental noise that computational methods cannot resolve. Additionally, the statistical robustness is constrained by the relatively small dataset size. While relatively sufficient for trend analysis, a larger experimental dataset would be required to further refine the quantitative correlation.

Therefore, the calculated Δ*G* values should be interpreted as qualitative indicators, effectively discriminating between strong binders and escape variants rather than serving as absolute quantitative predictors for subtle affinity differences. Although more rigorous approaches, such as umbrella sampling,^76^ Hamiltonian replica-exchange molecular dynamics (H-REMD)^77^ or accelerated weight histogram (AWH)^78^ can provide greater precision, their high computational demands make them impractical for a large set of antibody–RBD systems. It is worth noting that, while free energy perturbation (FEP) methods provide higher accuracy for predicting the impact of single-point mutations,^79,80^ applying them to heavily mutated variants such as KP.3 is computationally impractical. Consequently, the MM-PBSA approach remains the most effective strategy for systematically profiling the immune evasion potential across diverse viral lineages.

**Fig. 5:**
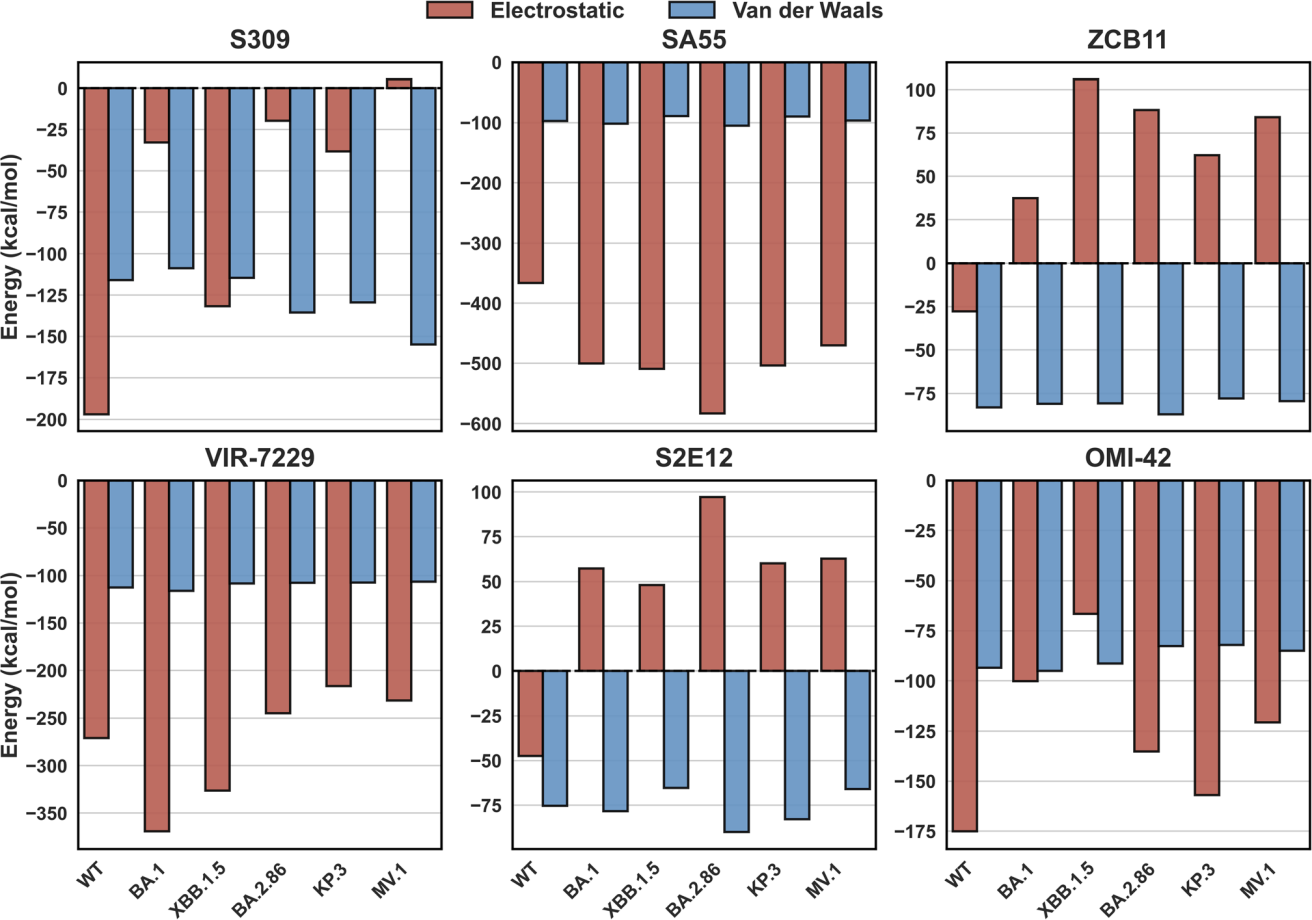
Decomposition of non-bonded interaction energy into electrostatic and van der Waals components across antibody classes. van der Waals interactions (blue bars) remain consistently favorable and almost constant across all variants, indicating preserved structural fit. In contrast, the electrostatic interactions (red bars) vary significantly: they remain highly attractive for broad neutralizers (SA55 and VIR-7229) but undergo a dramatic inversion from attractive to repulsive (positive value) for escape variants in the S2E12 and ZCB11 classes, identifying electrostatic repulsion as the primary determinant of immune evasion.

To decipher the physical origins of different interactions, we decomposed the total non-bonded energy into electrostatic and van der Waals components across all antibody classes (Fig. 5). This decomposition reveals a critical insight that supports our charge-centric hypothesis: while structural complementarity, which is represented by the vdW interaction, remains relatively stable across variants, the electrostatic interaction acts as the primary “switch” determining viral escape.

As shown in Fig. 5, the vdW contributions (blue bars) remain consistently favorable (negative) across all variants for all antibodies, suggesting that the mutations do not cause strong steric clashes that completely prevent physical contact. In stark contrast, the electrostatic profiles (red bars) exhibit drastic changes that dictate the binding outcome. This is most evident in class 1 (S2E12) and class 2 (ZCB11) antibodies. For these systems, the vdW terms remain favorable (about −80 kcal mol^−1^), but the electrostatic terms undergo a dramatic inversion from attractive (negative) in WT to repulsive (positive) in the Omicron variants. This explicitly quantifies that viral escape for these classes is driven not by loss of shape complementarity, but by the accumulation of positive charges on the RBD surface that electrostatically repel the positively charged antibodies.

Uniquely, the class 3 antibody S309 exhibits a hybrid behavior. Similar to the escape variants, S309 suffers a severe loss of electrostatic attraction, with the interaction flipping to a repulsive +5.31 kcal mol^−1^ in the MV.1 variant. However, unlike S2E12, which loses efficacy, S309 retains binding capacity. This is driven by the stabilization of *N*-glycans (N343 and N354), which act as molecular anchors to counteract the electrostatic repulsion at the protein interface. The detailed energetic decomposition of this glycan-mediated mechanism will be discussed in the subsequent subsection for class 3.

Conversely, broad neutralizers SA55 and VIR-7229 maintain their potency through massive electrostatic attraction (ranging from −500 to −300 kcal mol^−1^), which dominates the total non-bonded energy and overrides minor structural perturbations. These findings confirm that electrostatic remodeling, rather than steric hindrance alone, is the dominant evolutionary strategy for SARS-CoV-2 immune evasion. Guided by this charge-centric view, we dissected the specific mutational impacts and observed a hierarchical, two-tiered mechanism to modulate these energetic components.

### 3.3 Class 1: VIR-7229, S2E12, and OMI-42: electrostatic repulsion caused by mutations facilitates antibody evasion by SARS-CoV-2

**Fig. 6:**
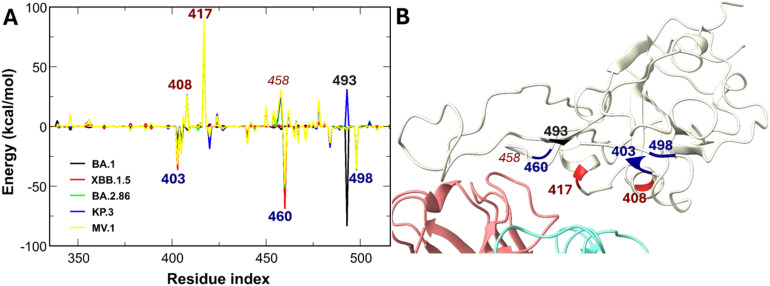
(A) Differences in non-bonded interaction energies between VIR-7229 and individual RBD residues of SARS-CoV-2 variants compared to the WT. Blue-labeled residues indicate mutations enhancing interactions by more than −25 kcal mol^−1^, red-labeled residues denote mutations weakening interactions by more than 25 kcal mol^−1^, and black-labeled residues represent mutated positions with variant-dependent energy contributions. (B) Schematic of the positions of the labeled RBD residues.

For the neutral antibody VIR-7229, binding is dominated by strong electrostatic attractions with RBD regions 400–500 (Fig. S4). In the Omicron subvariants, mutations slightly reduce binding affinity (Fig. 6A). The mutations R408S and K417N in all variants and Q493E in KP.3 weaken the interaction. Q493E causes electrostatic repulsion because the Q residue in the WT has about −3 kcal mol^−1^ energy with VIR-7229, but E has about +25 kcal mol^−1^. The R408S and K417N mutations disrupt the attractive electrostatic interaction. Mutations also reorient K458, reducing its contribution. On the other hand, mutations R403K, N460K, and Q498R improve the binding free energy between the RBD of the Omicron subvariants and the antibody, which rescues VIR-7229 from being evaded. These enhancing and weakening mutations are spatially proximate, with N460K located at the RBD and VIR-7229 interface (Fig. 6B). Additionally, N460K and Q498R are critical for human ACE2–RBD binding.^64^

Experimental results show that VIR-7229 exhibits a remarkably narrow escape profile with an escape mutation at position 456 of the RBD.^36^ In our simulations, although the interaction between residue 456 and VIR-7229 is weaker than those involving positively charged residues, its contribution to binding remains substantial, spanning from −17.12 kcal mol^−1^ in WT to approximately −11.72 in KP.3 and MV.1. This highlights the mutation of 456 from F to L/V in KP.3 and MV.1 significantly diminishes the energetic contribution of this residue. These findings underscore that key residues critical for human ACE2–RBD binding also bolster VIR-7229's potency against novel variants. We propose a tiered framework for these interactions: positively charged mutations at conserved sites represent tier 1, essential for preserving overall potency, while binding-region residues such as 456 constitute tier 2, providing supplementary affinity support.

**Fig. 7:**
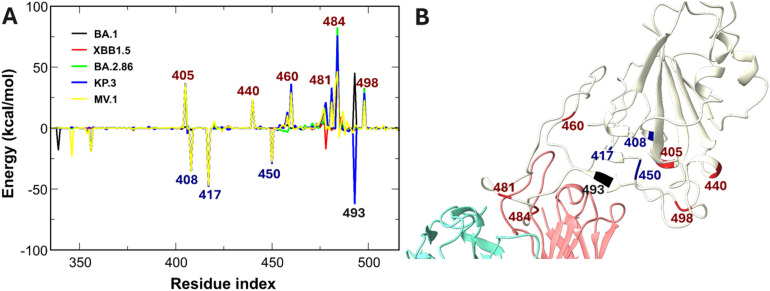
(A) Differences in non-bonded interaction energies between S2E12 and individual RBD residues of SARS-CoV-2 variants compared to the WT. Blue-labeled residues indicate mutations enhancing interactions by more than −25 kcal mol^−1^, red-labeled residues denote mutations weakening interactions by more than 25 kcal mol^−1^, and black-labeled residues represent mutated positions with variant-dependent energy contributions. (B) Schematic of the positions of the labeled RBD residues.

In stark contrast, the positively charged S2E12 (+3e) and OMI-42 exhibit high vulnerability to the same charge-increasing mutations that benefit VIR-7229 and ACE2 binding (Fig. 3). Experimental data show that S2E12 loses its potency against Omicron subvariants BA.5 and later,^56^ which is in agreement with our simulation results. R408S and K417N in RBD variants, Q493E in KP.3, and N450D in the RBD of BA.2.86, KP.3 and MV1 enhance the binding affinity, which is opposite to that observed in VIR-7229. Unfortunately, D405N, N440K, N460K, N481K, E484A/K, Q493R, and Q498R significantly increase the RBD net positive charge, generating severe electrostatic repulsion (Fig. 7). These disruptive mutations cluster near the S2E12-RBD interface (Fig. 7B) and also enhance RBD–human ACE2 interactions.^64^ Experimental evidence confirms that specific mutations, such as N460K, confer resistance to class 1 and 2 antibodies.^81^ Consequently, viral evolution renders S2E12 less effective, as mutations both improve receptor binding and facilitate escape from S2E12.

It is important to note that while van der Waals interactions provide a substantial and relatively constant attraction across variants, ranging from −116 to −65 kcal mol^−1^ for class 1 (Table S3), the electrostatic contribution exhibits much larger fluctuations and dictates the outcome of binding. For instance, in the high-affinity VIR-7229-BA.1 complex, the favorable electrostatic term (−369.22 kcal mol^−1^) is more than three times larger than the vdW term (−116.05 kcal mol^−1^), confirming the dominance of electrostatics in this potent antibody. Conversely, for S2E12-BA.1, the vdW interaction remains favorable (−78.39 kcal mol^−1^) due to structural fit, but the electrostatic term flips to a highly unfavorable value (+57.34 kcal mol^−1^) (Table S3). This highlights that, while vdW forces are essential for complex stability, electrostatic forces act as the primary factor determining susceptibility to viral escape or poor binding.

**Fig. 8:**
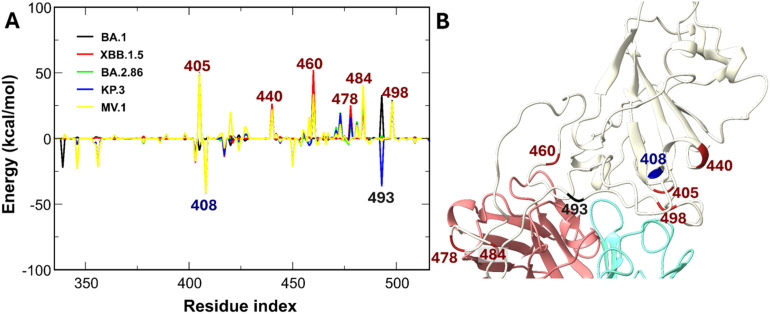
(A) Differences in non-bonded interaction energies between OMI-42 and individual RBD residues of SARS-CoV-2 variants compared to the WT. Blue-labeled residues indicate mutations enhancing interactions by more than −25 kcal mol^−1^, red-labeled residues denote mutations weakening interactions by more than 25 kcal mol^−1^, and black-labeled residues represent mutated positions with variant-dependent energy contributions. (B) Schematic of the positions of the labeled RBD residues.

Experiments indicate that OMI-42 and its derivative AZD3152 lose potency against variants EG.5.1 and KP.3.^58,61^ Our MM-PBSA calculations align with these findings, showing that OMI-42 exhibits significantly weaker binding free energies to KP.3 and MV.1 compared to the WT (Fig. 3). While the binding affinities for BA.1 and XBB.1.5 remain comparable to that for the WT, the BA.2.86 variant shows a marked reduction in affinity. Consistent with the tier 1 mechanism observed for S2E12, residue-level analysis confirms that charge-increasing mutations such as N460K and Q493R weaken OMI-42 interactions (Fig. 8), which explains the experimentally elevated IC50 values for BA.2 and BA.2.86.^63^ Crucially, OMI-42 is further compromised by tier 2 neutral mutations at position 456. Specifically, the F456L (KP.3) and F456V (MV.1) mutations destabilize the hydrophobic interface, shifting the interaction energy to unfavorable values of +1.0 and +3.6 kcal mol^−1^, respectively, in sharp contrast to the favorable −9.2 kcal mol^−1^ contribution of F456 in the WT. This finding is consistent with the MM-GBSA mutational profiles recently reported by Alshahrani *et al.*^82^ Experiments suggest that F456L substantially diminishes the affinity of OMI-42 and RBD.^61,74^ Collectively, our simulations suggest that the combination of electrostatic repulsion (tier 1) and hydrophobic disruption at residue 456 (tier 2) substantially impairs the binding affinity of OMI-42.

In summary, MM-PBSA analyses reveal that mutations N460K, Q493R, T478K, E484A/K, and Q498R in SARS-CoV-2 Omicron subvariant RBDs alter the electrostatic interactions, significantly affecting the binding energies of class 1 antibodies VIR-7229, S2E12, and OMI-42 to the RBD. These mutations also enhance RBD–human ACE2 binding,^64,83^ enabling SARS-CoV-2 variants to simultaneously strengthen receptor affinity and induce electrostatic repulsion to evade antibodies, particularly S2E12. The variant escape class 1 antibody follows a tiered framework: mutations altering the RBD's net charge most strongly impact antibody binding (tier 1), whereas neutral residues in the binding region, such as position 456, exert a secondary influence (tier 2).

### 3.4 Class 2 ZCB11: electrostatic repulsion driven by mutations promotes SARS-CoV-2 antibody evasion

Similar to S2E12, the class 2 antibody ZCB11 carries a high positive net charge (+4e), rendering it highly susceptible to the charge-increasing evolutionary trajectory of the RBD.^62^ Our MM-PBSA analysis confirms that ZCB11 exhibits significantly weaker binding free energies to XBB.1.5, BA.2.86, KP.3, and MV.1 compared to the WT (Fig. 3), a reduction driven principally by electrostatic repulsion (Table S3).

**Fig. 9:**
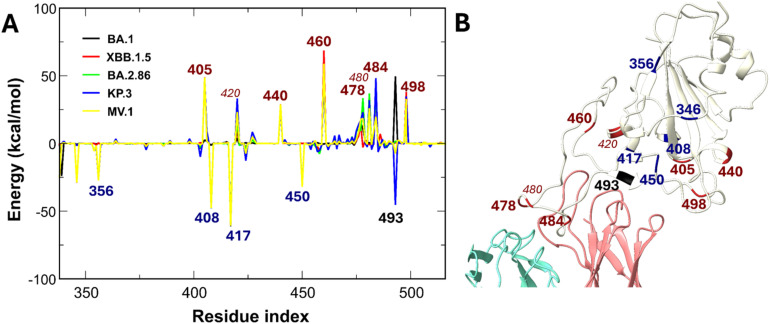
(A) Differences in non-bonded interaction energies between ZCB11 and individual RBD residues of SARS-CoV-2 variants compared to the WT. Blue-labeled residues indicate mutations enhancing interactions by more than −25 kcal mol^−1^, red-labeled residues denote mutations weakening interactions by more than 25 kcal mol^−1^, and black-labeled residues represent mutated positions with variant-dependent energy contributions. (B) Schematic of the positions of the labeled RBD residues. Residues 420 and 480 are not mutated.

Specific mutations T478K, Q493R, and Q498R, identified experimentally to affect ZCB11 potency, introduce positive charges at the critical 475–500 binding loop^34^ (Fig. 9). ZCB11 relies heavily on contact residues 477, 478, 487, and 460,^34^ but 478 is mutated to the positive charge residue K in all studied Omicron subvariants, and 460 is also replaced by K in XBB.1.5, BA.2.86, KP.3, and MV.1.^81^ Given the high positive charge of ZCB11, the N460K mutation generates a potent electrostatic penalty compared to BA.1 and WT, leading to positive electrostatic interaction energies (Fig. 9A). This repulsion is further compounded by interface-proximal mutations such as T478K, E484A, and Q498R (Fig. 9B). Consistently, experimental evidence confirms that charge-increasing mutations, such as A484K, confer resistance to class 1 and 2 antibodies.^81^ Although charge-reducing mutations, such as R408S, K417N, and N450D, theoretically enhance electrostatic attraction, their stabilizing contribution is overwhelmed by the stronger repulsive forces from the charge-increasing mutations. This arises partly because residues 478, 484, and 498 are positioned much closer to the antibody-binding interface. Consequently, the elevated positive charge of Omicron subvariant RBDs at the interface weakens ZCB11's binding, enabling evasion by XBB.1.5, BA.2.86, KP.3, and MV.1, consistent with experimental findings.^62^ This underscores that tier 1 mutations at the interface alone suffice to disrupt and evade class 2 antibodies such as ZCB11.

### 3.5 Class 3-S309: glycan-mediated compensatory stabilization counteracts protein interface mutations

Unlike class 1 and 2 antibodies, the class 3 antibody S309 is distinguished by its critical reliance on glycan interactions, as its epitope centers on the N343 glycosylation site. Our structural analysis confirms that the glycans maintain contact with S309 in 100% of the analyzed conformations (Fig. S3). Notably, BA.2.86 and its derivatives, such as KP.3 and MV.1, possess an additional glycan at N354 alongside N343. The experiments reported by de Campos-Mata *et al.* showed that S309 is evaded by BA.2.86 ^56^ (Table 1). However, Yang *et al.* indicated that S309 is still able to neutralize BA.2.86 and JN.1, although its binding affinities to these variants are decreased.^23^ Yang *et al.* observed that the interaction between glycans attached to N343 and N354 of RBD boosts the binding affinity between S309 and BA.2.86. Therefore, the inconsistencies between studies may stem from glycans attached to the RBD of novel variants such as BA.2.86 and KP.3.

We investigated the glycan contributions to S309-RBD binding and found significant roles in the WT and enhanced contributions in BA.2.86, KP.3, and MV.1 due to N354 glycan (Table 2). Glycans contribute approximately 33% to interface surface area in single-glycan variants (WT, BA.1, and XBB.1.5), rising to ∼50% in dual-glycan variants (Table 2).

**Table 2:** Contribution of *N*-glycans to the S309-RBD interface: total interface surface area (%) and decomposition of non-bonded interaction energies (kcal mol^−1^)

Variant		1 Glycan at N343			2 Glycans at N343 and N354		
		WT	BA.1	XBB.1.5	BA.2.86	KP.3	MV.1
Surface area (%)	Glycan	36.17	32.26	34.01	50.02	52.15	47.88
	Protein	63.83	67.74	65.99	49.98	47.85	52.12
Non-bonded energy (kcal mol^−1^)	Glycan	−87.15	−57.41	−81.70	−134.33	−130.53	−150.21
	Protein	−225.68	−84.24	−164.69	−21.05	−37.18	0.66

**Fig. 10:**
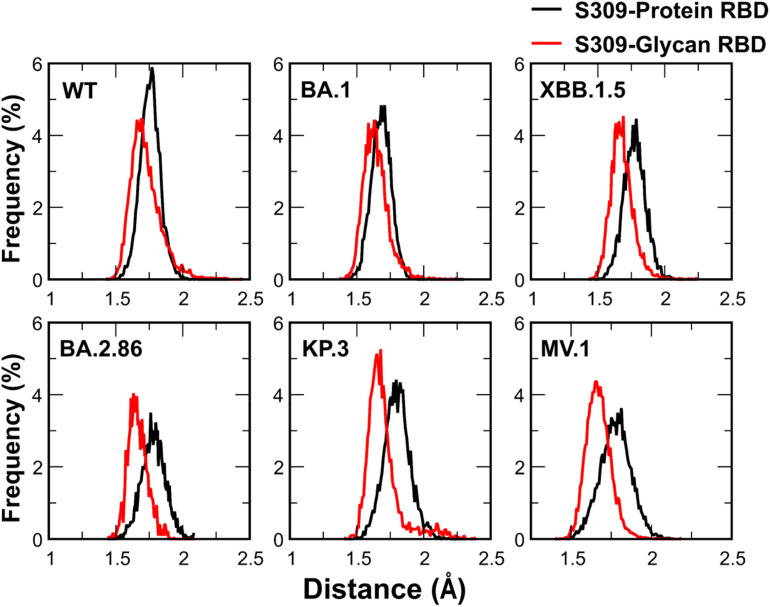
Probability distributions of the minimum distances between the S309 antibody and the protein moiety (black) *versus* the glycan moieties (red) of the SARS-CoV-2 RBD across six variants.

To characterize the spatial arrangement, we analyzed the distribution of the minimum distance between S309 and the RBD components (Fig. 10). These distributions exhibit distinct Gaussian-like peaks. For single-glycan variants (WT, BA.1, and XBB.1.5), the glycan-S309 distance peaks are consistently shifted toward shorter distances compared to the protein-S309 peaks, indicating that the glycan moiety approaches the antibody more closely than the protein surface. This proximity effect is amplified in dual-glycan variants (BA.2.86, KP.3, and MV.1), where the separation between the peaks is more pronounced. This confirms a shielding effect, where glycans mitigate the impact of underlying protein mutations. Consequently, the role of the glycans shifts from being supportive in earlier variants to becoming indispensable in MV.1. Our decomposition reveals that in the MV.1 complex, the protein–protein interface becomes electrostatically repulsive. However, the massive favorable energy contributed by *N*-glycans is sufficient to fully counteract this protein-level destabilization, effectively anchoring the antibody to the RBD and preserving binding affinity, despite the adverse mutations.

**Fig. 11:**
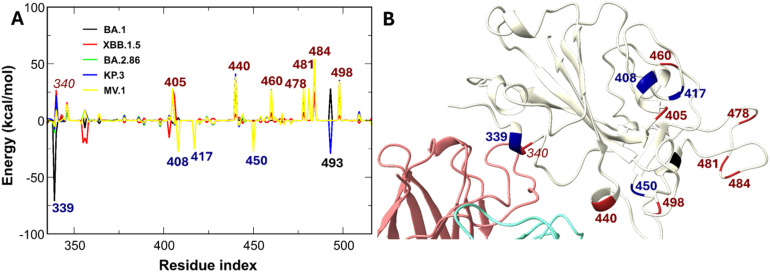
(A) Differences in non-bonded interaction energies between S309 and individual RBD residues of SARS-CoV-2 variants compared to the WT. Blue-labeled residues indicate mutations enhancing interactions by more than −25 kcal mol^−1^, red-labeled residues denote mutations weakening interactions by more than 25 kcal mol^−1^, and black-labeled residues represent mutated positions with variant-dependent energy contributions. (B) Schematic of the positions of the labeled RBD residues. Residue 340 is not mutated.

Regarding the protein interface, common charge-altering mutations (N460K, T478K, E484A, and Q498R) generally weaken binding (Fig. 11). A notable exception explains the weaker affinity of XBB.1.5 compared to BA.1. BA.1 possesses the G339D mutation, where the negative aspartate (D) electrostatically attracts the positive S309. The average non-bonded interaction energy between D339 in the BA.1 variant and S309 is −68.91 kcal mol^−1^, while the interaction with H339 in the XBB.1.5 variant is −4.98 kcal mol^−1^, and that of G339 in the WT variant is less favorable, at +1.72 kcal mol^−1^. Given the established enhancing role of glycans in S309-RBD interactions, the reduced contribution of glycans in the S309-XBB.1.5 complex results in a less negative binding free energy compared to both WT and BA.1. The region spanning amino acids 335–370 of the RBD exhibits strong interaction with the S309 antibody. Thus, while mutations in the 335–370 region act as tier 1 perturbations that impair protein affinity, S309 exhibits a unique counterbalancing mechanism: glycans stabilize the complex against these protein-level disruptions, preserving partial potency against highly mutated variants, consistent with experimental results.^23,51^

### 3.6 Class 4-SA55: electrostatic forces govern the stability of the SA55-RBD complex even in heavily mutated variants

The broad neutralization potency of SA55 is mechanistically explained by its electronegative character (−4e), which effectively mimics the electrostatic profile of ACE2. Because Omicron subvariants have more positively charged residues than WT, Omicron subvariants enhance their binding to human ACE2 *via* improvement of the electrostatic attractive interaction,^64,83,84^ inadvertently strengthening SA55 binding. Our MM-PBSA decomposition (Table S3) confirms that Omicron subvariants exhibit stronger electrostatic attraction to SA55 compared to WT, directly correlating with their increased net positive charge.

**Fig. 12:**
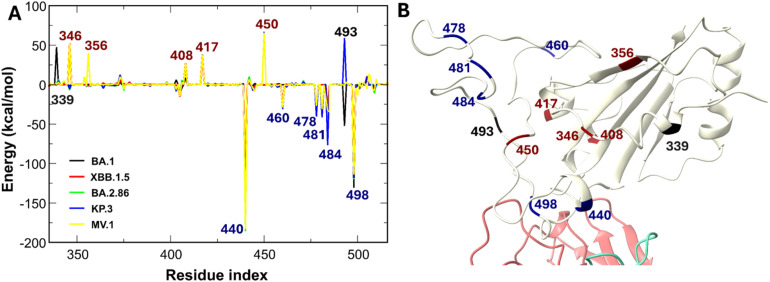
(A) Differences in non-bonded interaction energies between SA55 and individual RBD residues of SARS-CoV-2 variants compared to the WT. Blue-labeled residues indicate mutations enhancing interactions by more than −25 kcal mol^−1^, red-labeled residues denote mutations weakening interactions by more than 25 kcal mol^−1^, and black-labeled residues represent mutated positions with variant-dependent energy contributions. (B) Schematic of the positions of the labeled RBD residues.

Per-residue analysis (Fig. 12A) reveals that charge-increasing mutations, specifically N440K, N460K, T478K, N481K, E484A/K, and Q498R, universally enhance interaction energies. Specifically, residues 440 K and 498R are located near the interface of the RBD and SA55 (Fig. 12B and S3). Crucially, residues K440 and R498 are located directly at the SA55-RBD interface (Fig. 12B), acting as critical electrostatic anchors. While specific mutations, such as G339D (BA.1) or Q493E (KP.3), introduce local destabilization, their impact is structurally peripheral compared to the stabilizing effect of the interface-proximal Q498R. For instance, in KP.3, the enhanced electrostatic network formed by Q498R overrides the local repulsion of Q493E, allowing SA55 to maintain a tight binding free energy comparable to that of the WT (Fig. 3). To our knowledge, emerging variants such as LP.8.1 and NB.8.1 retain this critical N440K/Q498R motif, suggesting continued susceptibility to SA55. This mechanism is further validated by recent experimental work demonstrating that only artificial charge-reducing mutations (*e.g.*, G502D and G504D) allow pseudoviruses to escape SA55 neutralization.^85^ This finding aligns with our proposed tiered framework, wherein tier 1 mutations exert a strong influence on antibody–RBD binding affinities.

## 4. Conclusions

By integrating MD simulations and MM-PBSA analyses, we have elucidated the distinct binding and escape mechanisms of representative antibodies from four classes targeting the SARS-CoV-2 RBD by establishing a charge-centric hypothesis. This framework explicitly quantifies that electrostatic repulsion, rather than steric hindrance alone, is the primary driver determining the susceptibility of antibodies to viral escape. Through this thermodynamic lens, our results reveal a two-tiered evolutionary trend in viral evasion. Tier 1: the accumulation of charge-increasing mutations serves as the primary evasion mechanism. This strategy enhances affinity for the negatively charged ACE2 receptor while simultaneously generating severe electrostatic repulsion against positively charged antibodies, such as S2E12 (class 1) and ZCB11 (class 2), rendering them ineffective. Tier 2: the incorporation of nominally neutral mutations, such as F456L in KP.3, acts as a secondary mechanism. These mutations fine-tune evasion by disrupting local hydrophobic packing and destabilizing binding interfaces, as observed with OMI-42, even when electrostatic forces are not drastically altered. Notably, class 3 antibody S309 mitigates these electrostatic penalties by stabilizing glycan-mediated interactions, particularly at positions N343 and N354. Meanwhile, class 1 VIR-7229 and class 4 SA55 sustain efficacy by capitalizing on the viral evolutionary trajectory, leveraging the highly positively charged motifs that are acquired to enhance ACE2 binding. This highlights a critical vulnerability: antibodies with charge distributions opposite to ACE2 are easily evaded, whereas antibodies that mimic the electrostatic nature of ACE2 maintain broad neutralization. In brief, according to our charge-based hypothesis, for good binders, electrostatic interactions are more favorable than van der Waals interactions, whereas electrostatic interactions become less favorable for poor binders.

Our findings suggest that next-generation therapeutics should prioritize electrostatic resilience targeting epitopes with conserved electrostatic potentials that mimic ACE2 rather than solely focusing on shape complementarity. Furthermore, rational engineering strategies should exploit the glycan-binding motifs observed in S309 to develop constructs capable of resisting the charge-driven evolutionary trajectory of future SARS-CoV-2 variants.

## Author contributions

Hoang Linh Nguyen: conceptualization, investigation, formal analysis, writing – original draft, writing – review & editing. Nguyen Quoc Thai: formal analysis, writing – original draft, writing – review & editing. Linh Tran: formal analysis, writing – original draft, writing – review & editing. Mai Suan Li: conceptualization, writing – original draft, writing – review & editing.

## Conflicts of interest

The authors declare that there are no conflicts of interest.

## Supplementary Material

RA-016-D5RA08084C-s001

## Data Availability

The data supporting the results of this study are available in the supporting information (SI) and publicly accessible at https://doi.org/10.5281/zenodo.16394450. Our in-house program, developed for calculating the non-bonded interaction energy and entropy, is publicly accessible at https://doi.org/10.5281/zenodo.15910226. Supplementary information: Table S1: Physicochemical properties of the RBD binding epitopes for distinct antibody classes. The properties are calculated for the WT variant. Table S2: MM-PBSA results for the 100–200 ns, 100–400 ns, and 200–400 ns windows of the S309-WT RBD system. Errors represent standard deviations. Table S3: MM-PBSA results for antibody–RBD complexes of different viral variants (kcal mol^−1^). Errors represent standard deviations. Table S4: MM-PBSA results for S309-BA.1 RBD obtained from mutation of the WT (PDB ID 7R6W) (kcal mol^−1^). Errors represent standard deviations. Table S5: Δ*G* values obtained from MM-PBSA, experimental *K*_D_ values from Table 1, Δ*G*/*RT* (where *RT* = 0.5962 kcal mol^−1^ at *T* = 300 K), and natural logarithm of the experimental *K*_D_. Fig. S1: Root mean square deviation (RMSD) of C-alpha atoms obtained from six independent trajectories of antibody–RBD complexes of three WT RBD variants. Fig. S2: RMSD of C-alpha atoms of the S309-WT RBD system obtained from six independent trajectories extended to 400 ns. Fig. S3: Distribution of per-residue side-chain contacts of RBDs in complex with antibodies. Fig. S4: Heatmap of residue-level contact frequencies between RBD variants and the S2E12 antibody. Fig. S5: Heatmap of residue-level contact frequencies between RBD variants and the OMI-42 antibody. Fig. S6: Heatmap of residue-level contact frequencies between RBD variants and the ZCB11 antibody. Fig. S7: Heatmap of residue-level contact frequencies between RBD variants and the S309 antibody. Fig. S8: Heatmap of residue-level contact frequencies between RBD variants and the SA55 antibody. Fig. S9: Non-bonded interaction energy between antibodies and individual residues of the WT RBD. See DOI: https://doi.org/10.1039/d5ra08084c.
