# Supplementary material for: Exploring the diverse binding ability of SARS-CoV-2 variant RBDs to different antibody classes: a computational study
Source: RSC Adv. 2026 Mar 17;16(16):14761–77. doi: 10.1039/d5ra08084c (PMC12994731; doi:10.1039/d5ra08084c)
Supplement: RA-016-D5RA08084C-s001 [file RA-016-D5RA08084C-s001.pdf]

Supplementary Information (SI) for RSC Advances.
This journal is © The Royal Society of Chemistry 2026

Exploring Diverse Binding Ability of SARS-CoV-2 Variant RBDs to Different Antibody Classes: A Computational Study

Hoang Linh Nguyen[1,2,*], Nguyen Quoc Thai[3,4,*], Linh Tran[5,6,*], and Mai Suan Li[7,*]

[1]Institute of Fundamental and Applied Sciences, Duy Tan University, Ho Chi Minh City 70000, Vietnam

[2]Faculty of Environmental and Natural Sciences, Duy Tan University, Da Nang 50000, Vietnam

[3]Dong Thap University, 783 Pham Huu Lau Street, Ward 6, Cao Lanh City, Dong Thap 81000, Vietnam

[4] Faculty of Physics, VNU University of Science, Vietnam National University, 334 Nguyen Trai, Hanoi 10000, Vietnam

[5]University of Health Sciences, Vietnam National University Ho Chi Minh City, Ho Chi Minh City 70000, Vietnam

[6]Research Center for Discovery and Development of Healthcare Products, Vietnam National University Ho Chi Minh City, Ho Chi Minh City 70000, Vietnam

[7]Institute of Physics, Polish Academy of Sciences, al. Lotnikow 32/46, 02-668, Warsaw, Poland

*Email: nguyenhoanglinh9@duytan.edu.vn, nqthai@dthu.edu.vn, tttlinh@uhsvnu.edu.vn, masli@ifpan.edu.pl

SUPPORTING INFORMATION

Table S1: Physicochemical properties of the RBD binding epitopes for distinct antibody classes. The properties are calculated for WT variant.

Antibody Class	Number of Contact Residues	Buried Surface Area ($Å^2$)	Epitope Net Charge (e)	Total hydrophobicity[1]	Mean hydrophobicity
Class 1 (VIR-7229)	24	871.1	3	-43.5	-1.8
Class 2 (ZCB11)	17	726.1	1	-14.5	-0.9
Class 3 (S309)	19	550.8	2	-14.0	-0.7
Class 4 (SA55)	21	798.4	0	-21.2	-1.0

Table S2: MM-PBSA results for the 100–200 ns, 100–400 ns, and 200–400 ns windows of the S309-WT RBD system. Errors represent standard deviations.

Time window	Electrostatic	Van der Waals	Polar solvation	Non-polar solvation	Entropy	ΔG
WT 100-200 ns	-196.93 ± 16.04	-115.90 ± 5.45	255.99 ± 17.53	-18.79 ± 1.22	41.85 ± 6.93	-33.79 ± 4.63
WT 100-400 ns	-201.37 ± 15.06	-116.69 ± 4.16	261.85 ± 17.65	-19.05 ± 1.19	43.51 ± 2.61	-31.74 ± 4.90
WT 200-400 ns	-202.52 ± 17.17	-117.96 ± 6.48	264.35 ± 16.22	-19.20 ± 1.23	44.84 ± 2.85	-30.49 ± 5.62

Table S3: MM-PBSA results for antibodies-RBDs of viral variants (kcal/mol). Errors represent standard deviations.

Antibody	Viral variant	Electrostatic	Van der Waals	Polar solvation	Non-polar solvation	Entropy	ΔG
S309	WT	-196.93 ± 16.04	-115.90 ± 5.45	255.99 ± 17.53	-18.79 ± 1.22	41.85 ± 6.93	-33.79 ± 4.63
	BA.1	-32.84 ± 3.63	-108.81 ± 9.45	80.02 ± 19.48	-17.70 ± 1.60	45.15 ± 2.35	-34.17 ± 6.41
	XBB.1.5	-131.76 ± 14.11	-114.63 ± 6.50	197.21 ± 21.53	-18.29 ± 0.80	54.24 ± 8.80	-13.23 ± 6.24
	BA.2.86	-19.80 ± 5.27	-135.58 ± 13.09	112.46 ± 28.83	-21.77 ± 1.83	45.22 ± 6.21	-19.47 ± 5.93
	KP.3	-38.31 ± 10.61	-129.40 ± 10.28	134.70 ± 21.36	-21.47 ± 2.72	40.77 ± 6.40	-13.71 ± 7.20
	MV.1	5.31 ± 7.53	-154.86 ± 14.64	111.06 ± 23.70	-24.98 ± 2.45	52.95 ± 12.37	-10.53 ± 2.02
SA55	WT	-366.54 ± 26.31	-97.09 ± 8.71	422.30 ± 31.06	-14.57 ± 1.24	37.11 ± 3.22	-18.80 ± 3.42
	BA.1	-500.50 ± 52.85	-101.55 ± 8.38	549.98 ± 52.03	-15.75 ± 1.46	44.61 ± 6.35	-23.21 ± 4.50
	XBB.1.5	-509.32 ± 33.31	-89.01 ± 9.49	554.71 ± 36.41	-13.46 ± 1.61	37.12 ± 5.26	-19.96 ± 4.59
	BA.2.86	-583.31 ± 33.44	-104.88 ± 8.74	644.46 ± 38.62	-16.67 ± 1.43	40.03 ± 4.34	-20.37 ± 2.04
	KP.3	-504.00 ± 42.12	-89.84 ± 8.92	554.45 ± 41.77	-13.90 ± 1.44	36.69 ± 2.65	-16.60 ± 5.40
	MV.1	-470.22 ± 35.27	-96.34 ± 14.80	520.87 ± 42.58	-14.90 ± 2.97	37.64 ± 2.39	-22.96 ± 6.96
ZCB11	WT	-27.79 ± 5.73	-82.98 ± 9.47	73.71 ± 5.64	-13.48 ± 1.19	33.21 ± 2.54	-17.33 ± 4.12
	BA.1	37.41 ± 5.17	-80.92 ± 7.58	16.20 ± 4.36	-12.28 ± 0.70	29.65 ± 3.80	-9.94 ± 6.29
	XBB.1.5	105.98 ± 16.24	-80.67 ± 9.01	11.56 ± 6.97	-12.57 ± 1.33	33.47 ± 2.25	57.77 ± 7.36
	BA.2.86	88.33 ± 15.80	-86.99 ± 7.06	26.75 ± 6.26	-13.30 ± 0.84	34.49 ± 3.96	49.28 ± 14.62
	KP.3	62.19 ± 16.46	-77.86 ± 5.23	23.64 ± 12.18	-11.27 ± 0.53	34.42 ± 3.39	31.12 ± 11.54
	MV.1	84.20 ± 11.48	-79.39 ± 8.37	6.87 ± 4.41	-11.71 ± 0.69	35.22 ± 3.78	35.20 ± 8.02
VIR-7229	WT	-270.97 ± 10.89	-112.65 ± 2.42	345.46 ± 12.95	-16.36 ± 0.21	33.95 ± 2.13	-20.55 ± 3.10
	BA.1	-369.22 ± 22.83	-116.05 ± 7.41	436.66 ± 23.94	-17.09 ± 0.81	38.14 ± 3.81	-27.57 ± 4.88

	XBB.1.5	-326.27 ± 12.22	-108.30 ± 4.96	381.91 ± 13.72	-15.88 ± 0.43	35.55 ± 2.79	-33.09 ± 5.61
	BA.2.86	-244.92 ± 25.89	-107.64 ± 4.27	299.93 ± 29.04	-15.53 ± 0.56	37.62 ± 2.69	-30.54 ± 2.91
	KP.3	-216.48 ± 20.39	-107.42 ± 6.14	280.54 ± 24.16	-16.09 ± 0.70	38.23 ± 3.89	-21.22 ± 4.13
	MV.1	-231.42 ± 44.08	-106.32 ± 9.99	296.22 ± 47.76	-16.19 ± 1.17	41.46 ± 3.75	-16.24 ± 4.82
	WT	-47.46 ± 7.40	-75.34 ± 3.50	93.92 ± 6.43	-11.94 ± 0.43	32.34 ± 3.24	-8.48 ± 3.41
	BA.1	57.34 ± 17.89	-78.39 ± 4.68	30.53 ± 9.01	-11.66 ± 0.39	32.92 ± 2.45	30.75 ± 10.46
	XBB.1.5	48.14 ± 10.44	-65.27 ± 4.73	22.11 ± 5.03	-10.70 ± 0.65	28.89 ± 3.73	22.91 ± 5.24
S2E12	BA.2.86	97.12 ± 34.75	-90.03 ± 11.23	13.33 ± 7.28	-13.29 ± 1.60	35.17 ± 2.92	42.30 ± 27.42
	KP.3	60.24 ± 24.85	-82.83 ± 7.75	29.17 ± 14.00	-12.25 ± 1.32	31.40 ± 2.80	25.73 ± 11.03
	MV.1	62.80 ± 25.77	-65.90 ± 12.69	19.88 ± 11.62	-10.42 ± 1.81	32.79 ± 3.45	39.14 ± 24.38
	WT	-175.03 ± 14.34	-93.52 ± 3.93	232.73 ± 9.89	-14.79 ± 0.47	32.38 ± 1.12	-18.23 ± 4.20
	BA.1	-100.17 ± 15.94	-95.01 ± 2.29	158.42 ± 15.03	-15.04 ± 0.36	34.01 ± 2.58	-17.79 ± 5.77
	XBB.1.5	-66.50 ± 10.49	-91.33 ± 4.70	121.45 ± 11.89	-14.28 ± 0.83	34.24 ± 3.05	-16.42 ± 4.67
OMI-42	BA.2.86	-135.19 ± 28.02	-82.63 ± 5.87	189.89 ± 20.16	-13.23 ± 1.03	33.42 ± 2.55	-7.75 ± 3.02
	KP.3	-156.93 ± 27.68	-82.03 ± 10.70	215.69 ± 22.84	-13.19 ± 1.64	35.43 ± 3.04	-1.04 ± 8.10
	MV.1	-120.66 ± 66.48	-85.00 ± 12.89	187.46 ± 71.37	-13.97 ± 1.93	33.65 ± 2.16	1.48 ± 7.87

Table S4: MM-PBSA results for S309-BA.1 RBD obtained from mutation of WT (PDB id 7R6W) (kcal/mol). Errors represent standard deviations.

System	Electrostatic	Van der Waals	Polar solvation	Non-polar solvation	Entropy	ΔG
Modeled BA.1 system from 7R6W	-148.35 ± 14.10	-114.71 ± 4.56	202.47 ± 19.90	-19.15 ± 0.99	45.40 ± 2.47	-34.33 ± 2.87
BA.1 system with PDB 7YAD	-32.84 ± 3.63	-108.81 ± 9.45	80.02 ± 19.48	-17.70 ± 1.60	45.15 ± 2.35	-34.17 ± 6.41

Table S5: ΔG obtained from MM-PBSA, experimental KD values from Table 1, ΔG/RT where RT = 0.5962 kcal/mol at T = 300 K, and natural logarithm of experimental KD.

System	ΔG(calc)	KD(exp)	ΔG/RT	ln(KD)
S309-WT	-33.79	0.88* (0.2–2.21)**	-56.67	-0.13* (-1.61 – -0.21)*
S309-BA.1	-34.17	6.7 (0.9–10.98)	-57.31	1.90 (-0.11 – 2.39)
S309-XBB.1.5	-13.23	1.6	-22.19	0.47
SA55-WT	-18.80	0.25 (0.14–0.36)	-31.53	-1.39 (-1.97– -1.02)
SA55-XBB.1.5	-19.96	0.81 (0.72–0.89)	-33.48	-0.22 (-0.33–-0.12)
SA55-BA.2.86	-20.37	0.12	-34.17	-2.12
SA55-KP.3	-16.60	0.13 (0.04–0.22)	-27.84	-2.04(-3.22–-1.51)
ZCB11-WT	-17.33	0.15	-29.07	-1.90
ZCB11-BA.1	-9.94	0.14	-16.67	-1.97
VIR-7229-WT	-20.55	0.2	-34.47	-1.6
VIR-7229-BA.1	-27.57	0.4	-46.24	-0.9
VIR-7229-XBB.1.5	-33.09	0.1	-55.50	-2.3
VIR-7229-BA.2.86	-30.54	0.3	-51.22	-1.2
S2E12-WT	-8.48	1.06 (0.02–2.1)	-14.22	0.06 (-3.91–0.74)
S2E12-BA.1	30.75	41.65 (39.3–44)	51.58	3.73 (3.67–3.78)
OMI-42-WT	-18.23	0.46 (0.25–0.678)	-30.58	-0.77 (-1.39– -0.39
OMI-42-BA.1	-17.79	0.24	-29.84	-1.43
OMI-42-XBB.1.5	-16.42	0.32 (0.21–0.45)	-27.54	-1.15 (-1.56– -0.80)
OMI-42-BA.2.86	-7.75	0.4 (0.36–0.44)	-13.00	-0.92 (-1.02– -0.82)

*mean
**range

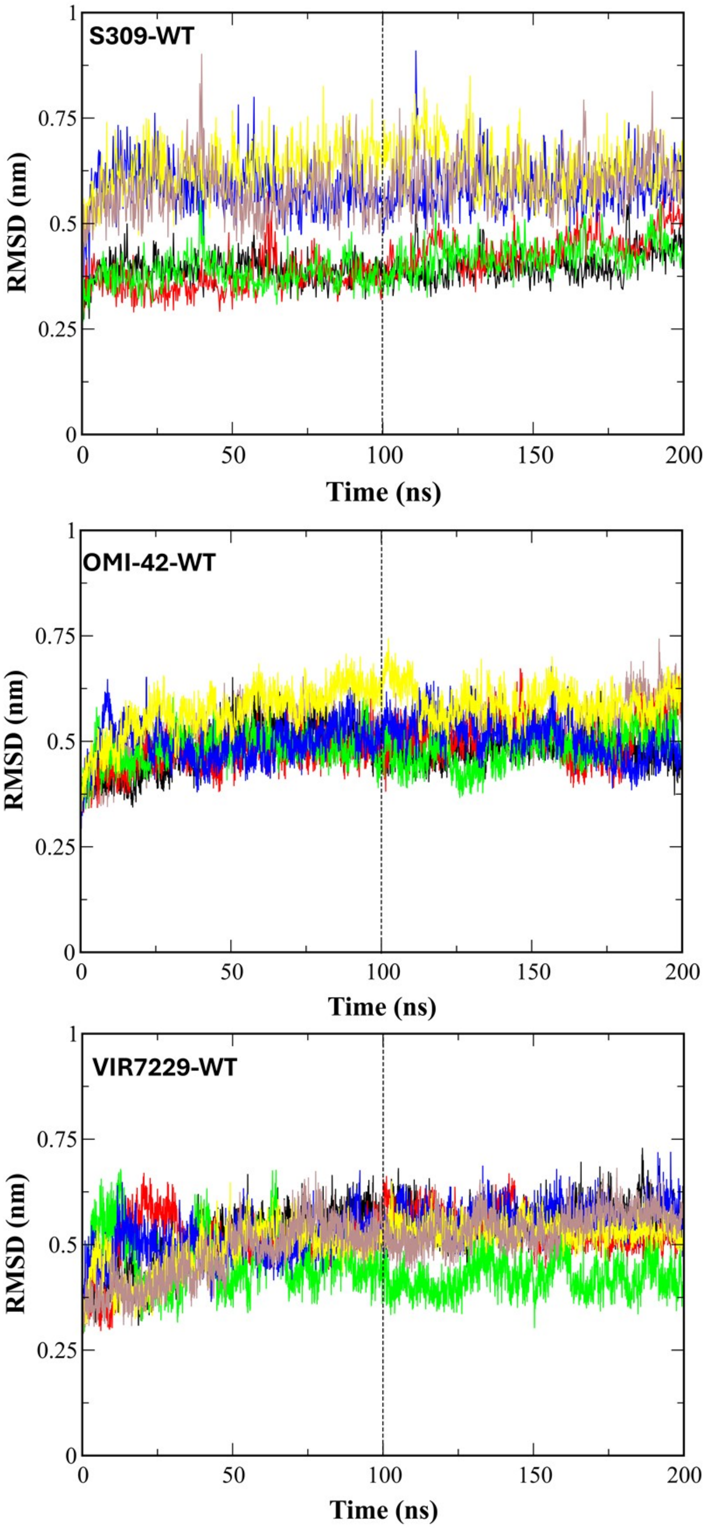

Figure S1: Time dependence of the root mean square deviation (RMSD) of C-alpha atoms obtained from six independent trajectories for the antibody-RBD complex of three WT RBD variants. The dotted line marks the time t = 100 ns when the system reaches equilibrium.

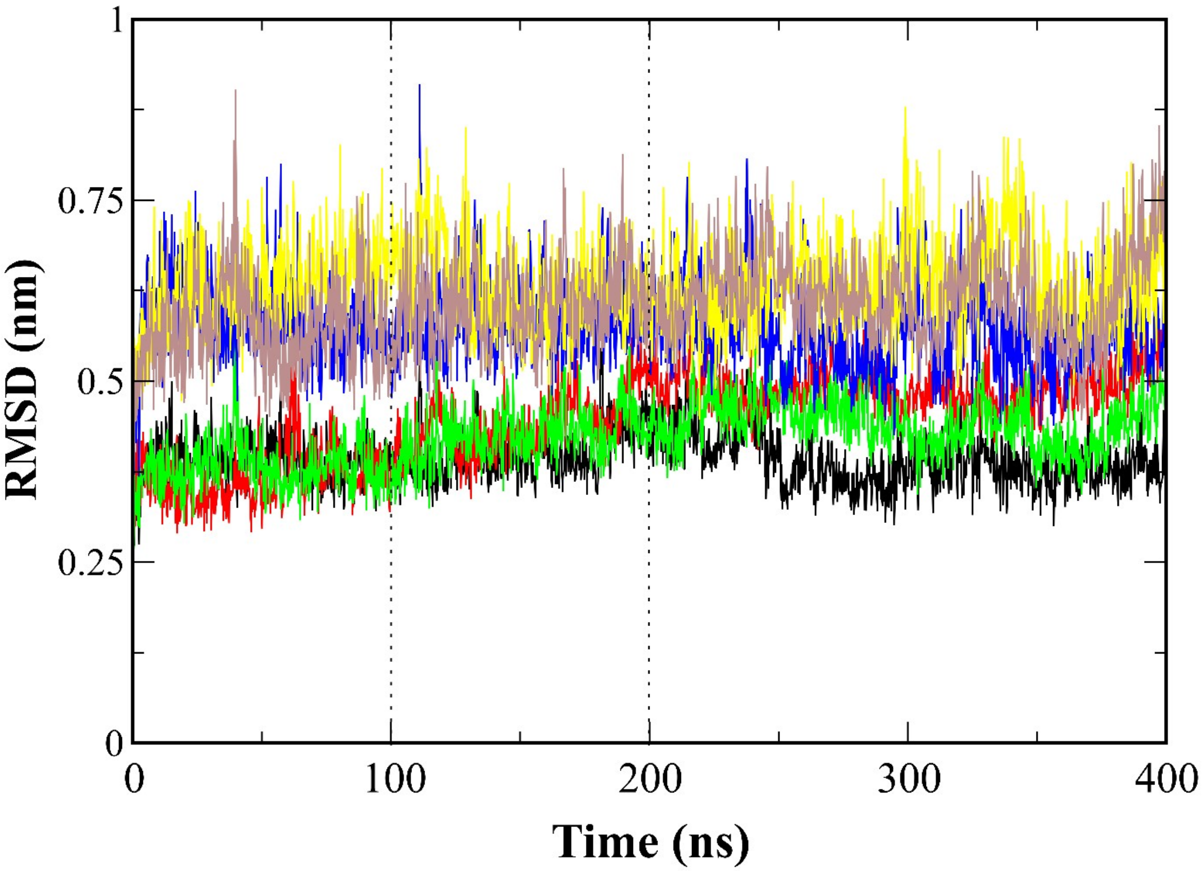

Figure S2: Time dependence of RMSD of C-alpha atoms of the S309-WT RBD complex obtained from six independent trajectories extended to 400 ns.

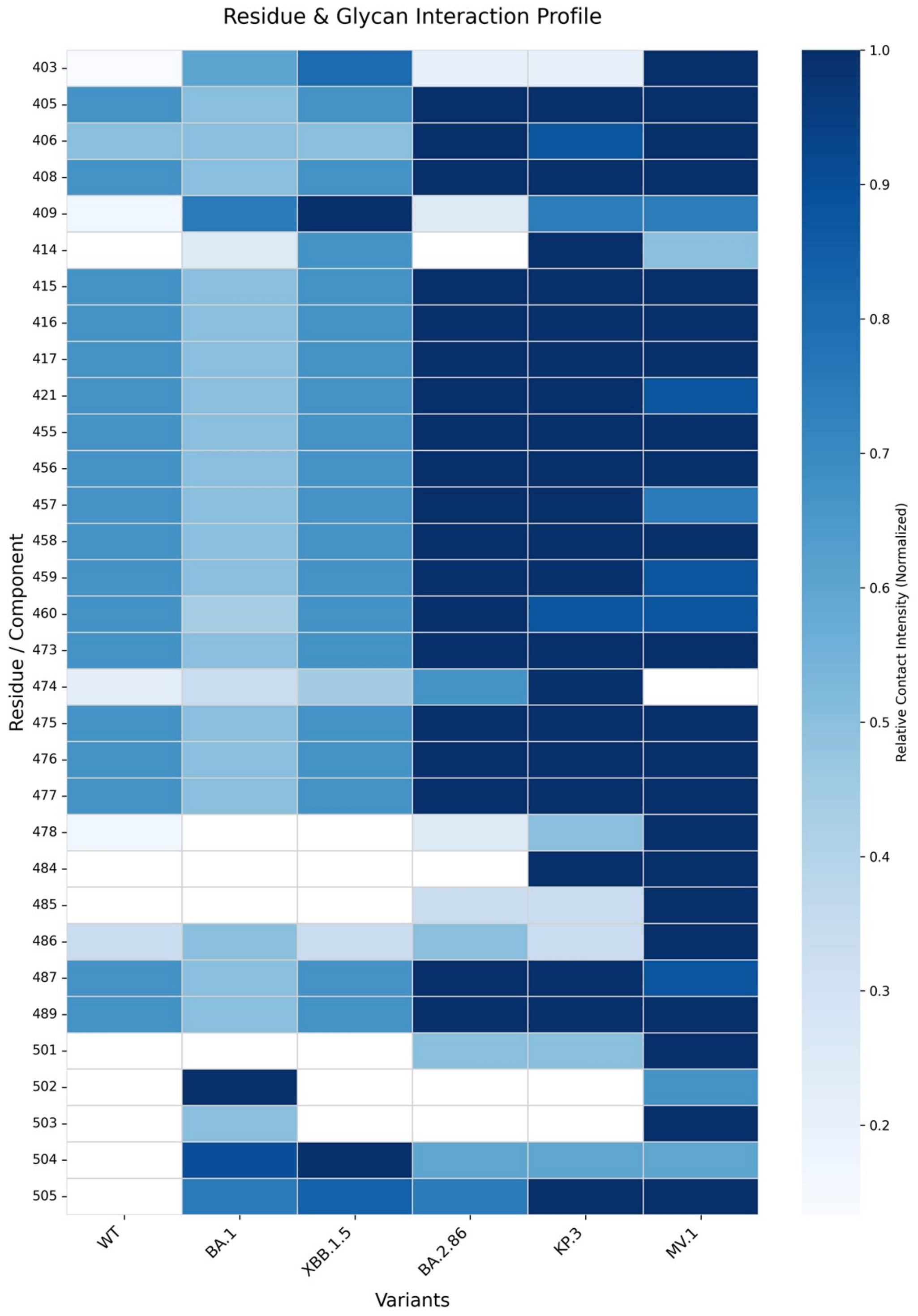

Figure S3: Heatmap of residue-level contact frequencies between RBD variants and the VIR-7229 antibody.

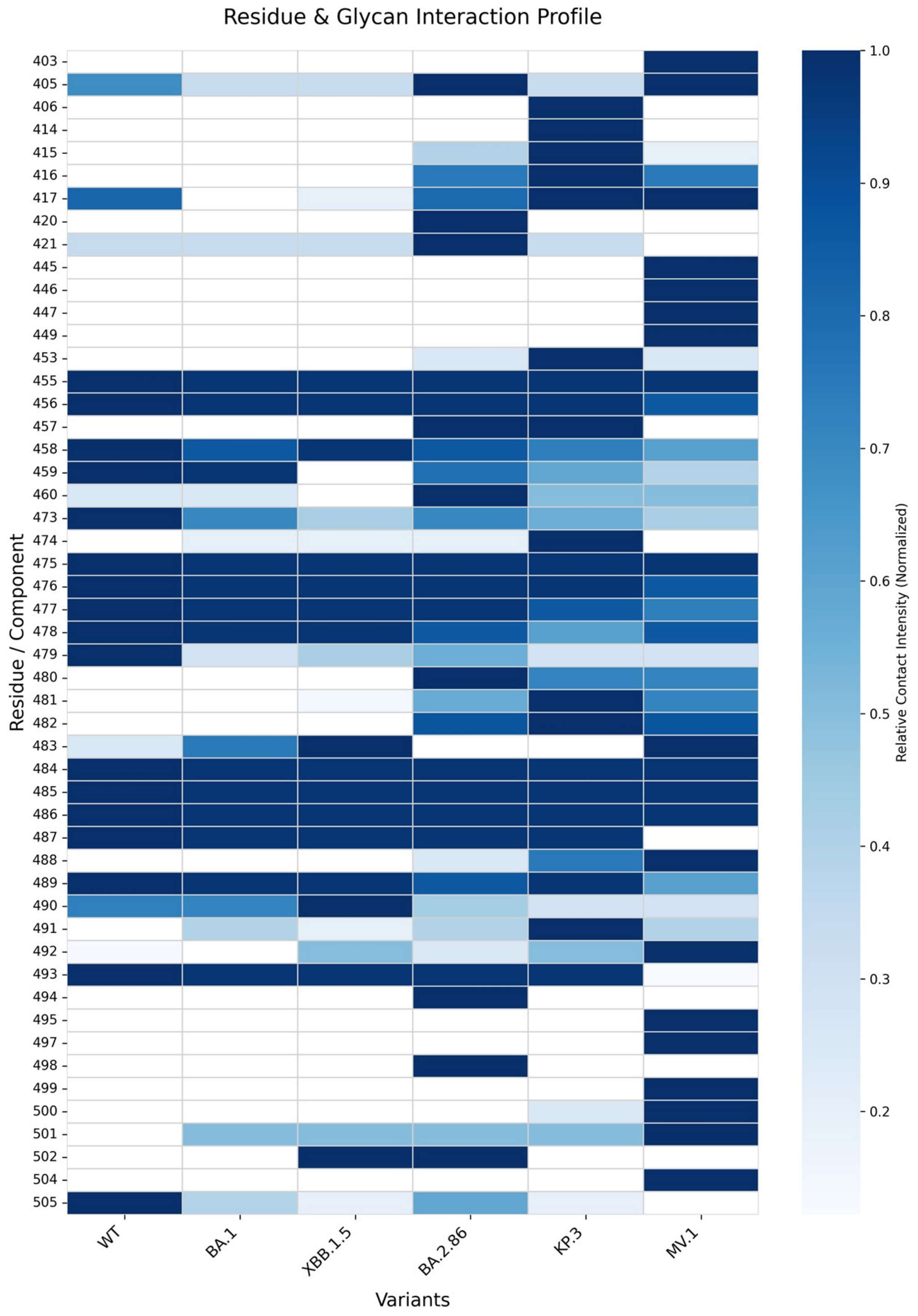

Figure S4: Heatmap of residue-level contact frequencies between RBD variants and the S2E12 antibody.

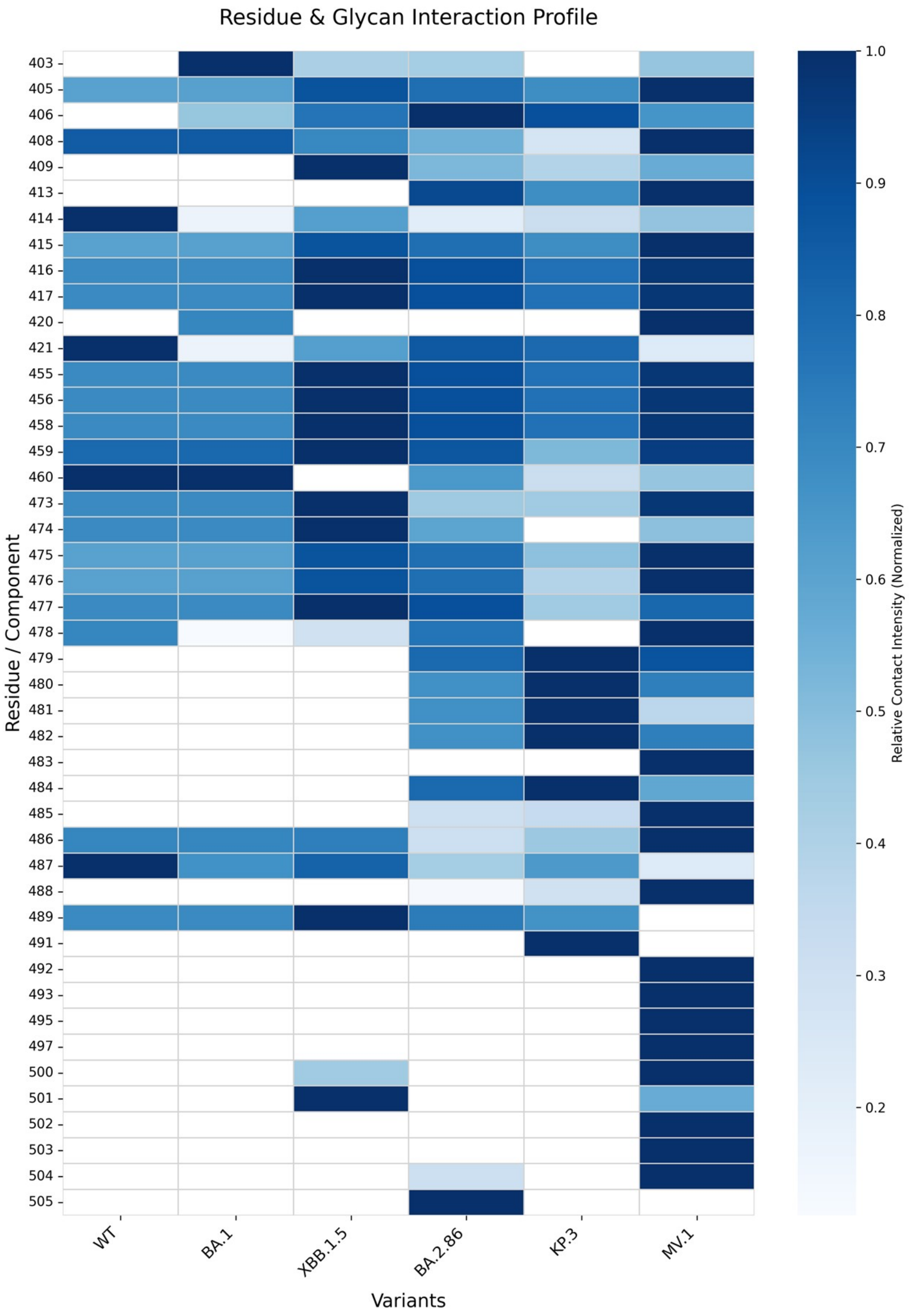

Figure S5: Heatmap of residue-level contact frequencies between RBD variants and the OMI-42 antibody.

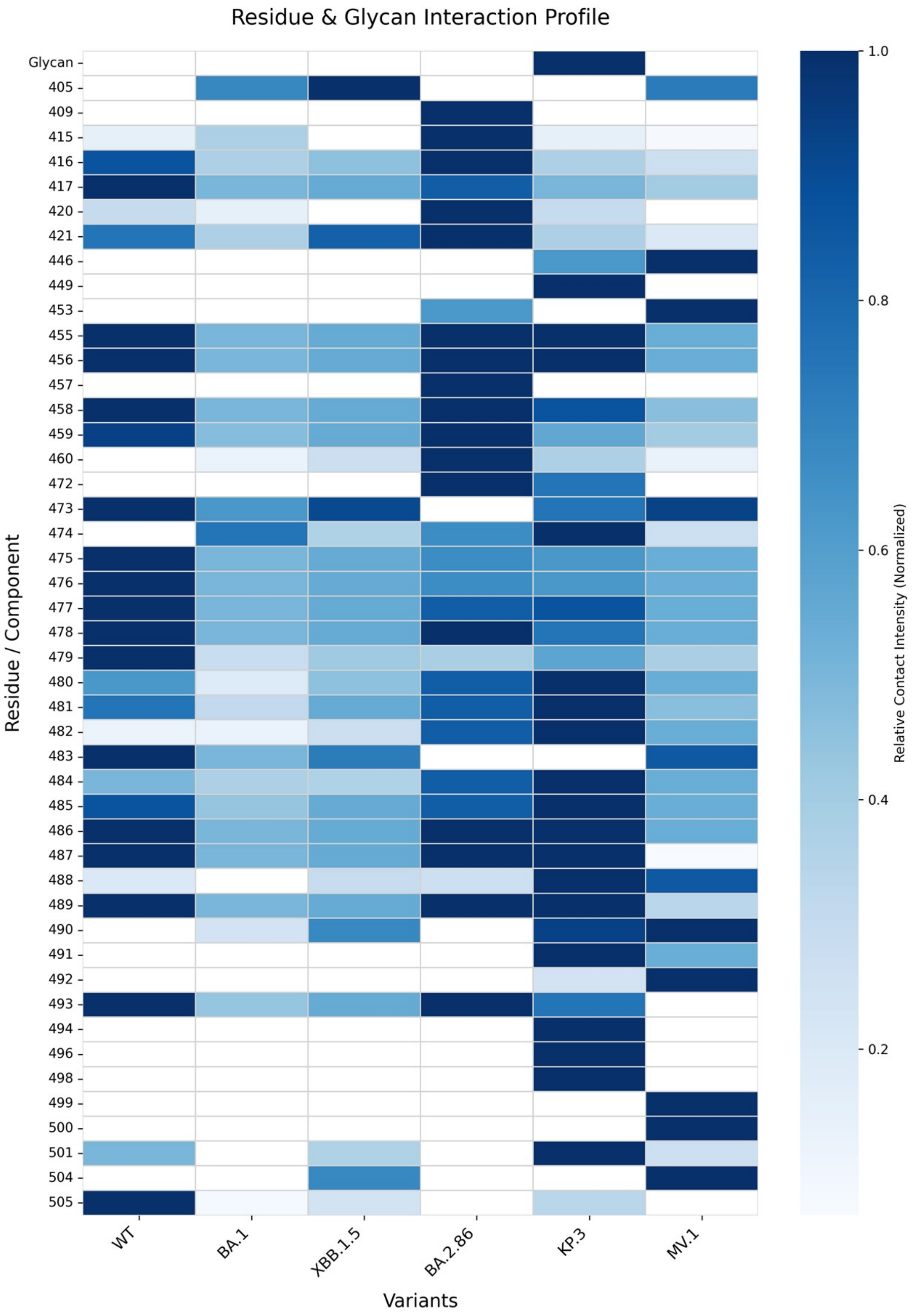

Figure S6: Heatmap of residue-level contact frequencies between RBD variants and the ZCB11 antibody.

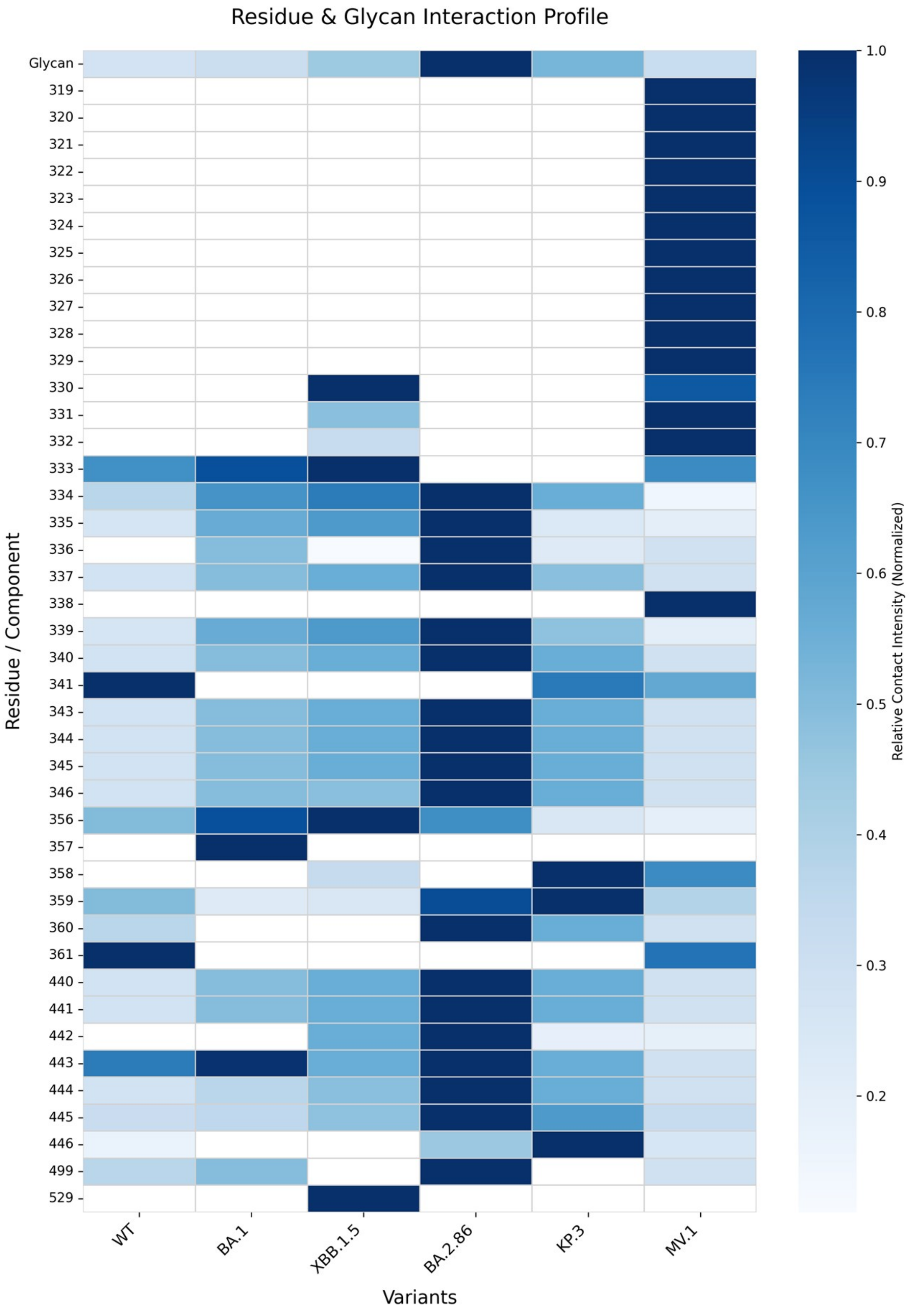

Figure S7: Heatmap of residue-level contact frequencies between RBD variants and the S309 antibody.

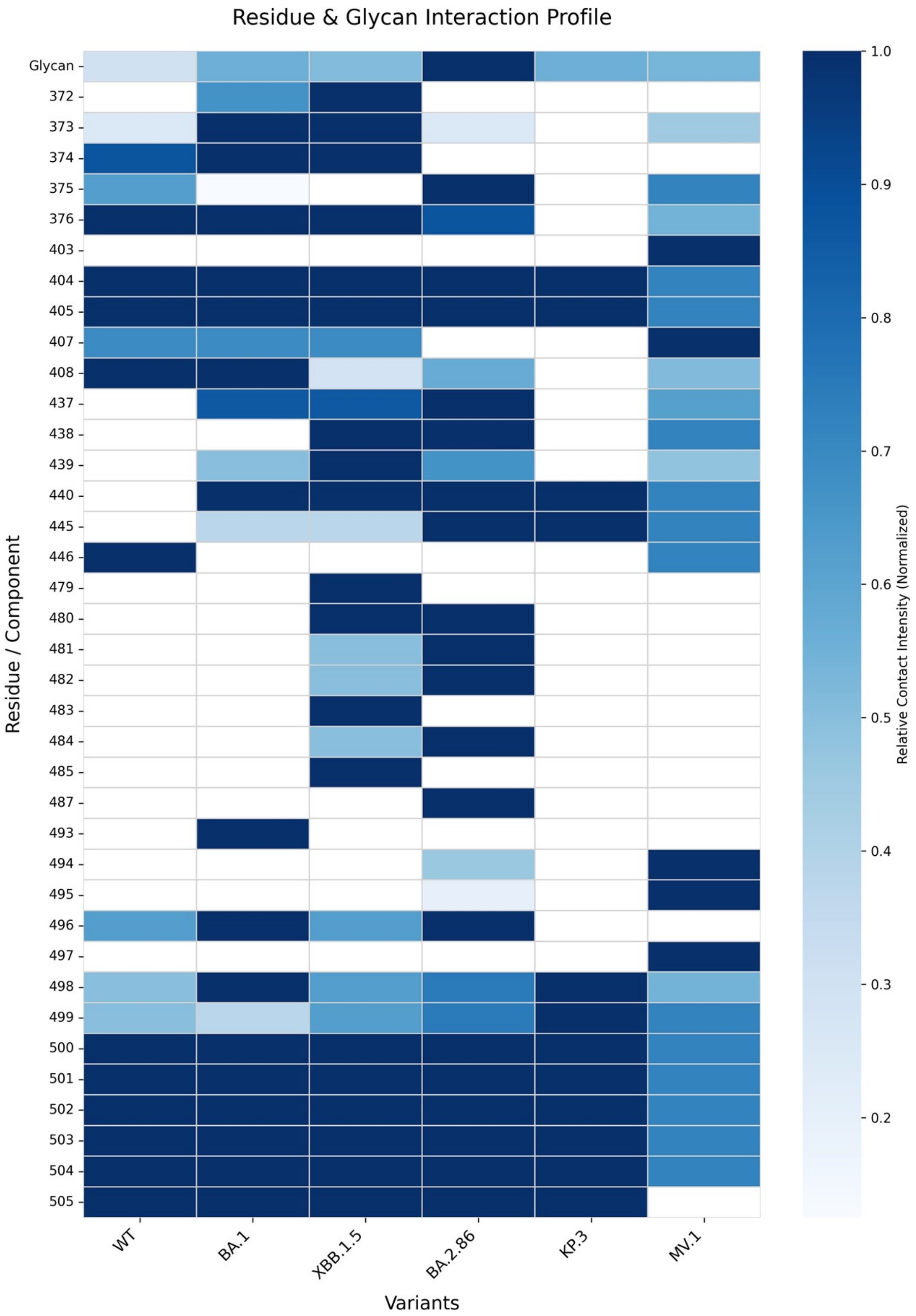

Figure S8: Heatmap of residue-level contact frequencies between RBD variants and the SA55 antibody.

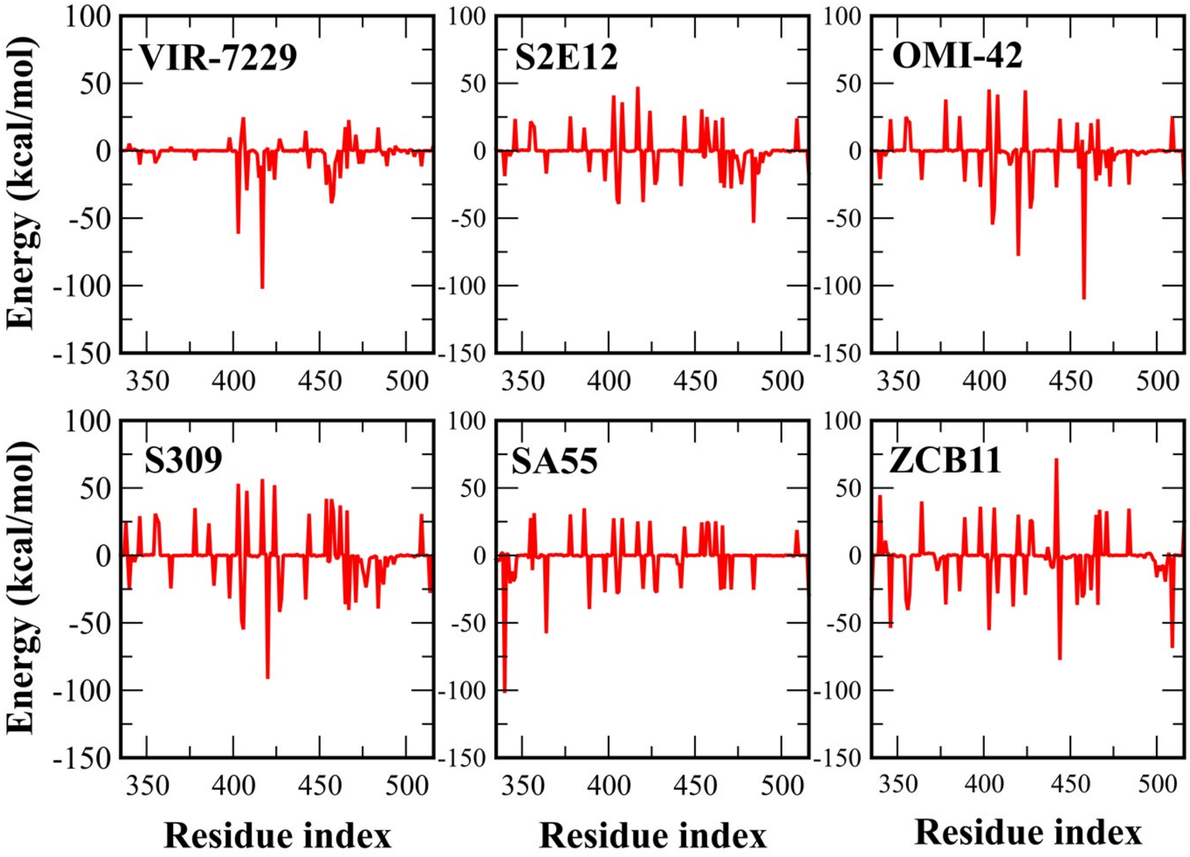

Figure S9: non-bonded interaction energy between antibodies and individual residues of WT RBD.

(1) Kyte, J.; Doolittle, R. F. A simple method for displaying the hydropathic character of a protein. *J Mol Biol* **1982**, *157* (1), 105–132. DOI: 10.1016/0022-2836(82)90515-0
